# Supplementary material for: Relation of in-utero exposure to antiepileptic drugs to pregnancy duration and size at birth
Source: PLoS One. 2019 Aug 5;14(8):e0214180. doi: 10.1371/journal.pone.0214180 (PMC6681941; doi:10.1371/journal.pone.0214180)
Supplement: S1 File — (DOCX) [file pone.0214180.s001.docx]

**Systematic literature search**

This is supporting information to:

Relation of in-utero exposure to antiepileptic drugs to pregnancy duration and size at birth

Margulis AV, Hernandez-Diaz H, McElrath T, Rothman KJ, Plana E, Almqvist C, D´Onofrio BM, Oberg AS

Table of Contents

[Abbreviations 1](#_Toc520744957)

[1 Introduction 2](#_Toc520744958)

[2 Methods 2](#_Toc520744959)

[2.1 Eligibility criteria 2](#_Toc520744960)

[2.2 Literature search strategy and terms 3](#_Toc520744961)

[2.3 Screening process, selection of studies and data extraction 5](#_Toc520744962)

[2.4 Statistical analysis 5](#_Toc520744963)

[3 Results 5](#_Toc520744964)

[3.1 Literature search and screening process 5](#_Toc520744965)

[3.2 Extracted data 7](#_Toc520744966)

[4 References 8](#_Toc520744967)

Abbreviations

| AED | antiepileptic drug |
| --- | --- |
| ATC | Anatomic Therapeutic Classification |
| HELLP | hemolysis, elevated liver enzymes and low platelet count |
| ICPE | International Conference on Pharmacoepidemiology & Therapeutic Risk Management |
| p | percentile |
| SD | standard deviation |
| SGA | small for gestational age |

# Introduction

We performed a systematic literature search to retrieve available information on the association between the use of individual antiepileptic drugs (AEDs) in pregnancy and duration of pregnancy, birth weight, birth length, and head circumference. The objectives were (1) to find published evidence to support the introduction and discussion of this manuscript and (2) to support the selection of a reference drug for analysis for this study. Additionally, we sought to develop an abstract for the International Conference on Pharmacoepidemiology & Therapeutic Risk Management (ICPE) 2017 describing the work toward objective 2, which was presented as a poster.[^1^](#_ENREF_1)

# Methods

## Eligibility criteria

1. Study eligibility criteria

| Study eligibility criteria |
| --- |
| Initial criteria |
| - Dates: no limits. - Languages: no limits for search. - Publication types: original research. - Study type: prospective or retrospective, observational or interventional, not case reports or case series. - Exposure, outcomes, and statistical methods: must present adjusted relative estimates of the effect of individual AEDs on duration of pregnancy, birth weight, length at birth or head circumference at birth, treated as continuous or categorical variables. No restrictions are placed for the reference with regard to exposure (i.e., eligible reference groups will include unexposed women, women exposed to other AEDs, women with or without epilepsy or other conditions). Reasons to include only results on individual drugs are that clinical practice changed over time and the group of combined AEDs represents different drug mixes over time and that a systematic literature review that explored the effect of combined AEDs was published recently.[^2^](#_ENREF_2) |
| Refinement during level 1 screening |
| - If study outcomes are not presented in the abstract, articles must include in the title or abstract language denoting that findings on the outcomes of interest for this literature search are provided in the full text. - Studies focusing on hemolysis, elevated liver enzymes, and low platelet count (HELLP) syndrome; pre-eclampsia; eclampsia; or tocolysis will be excluded because this literature search is oriented towards chronic treatment with AEDs, including the possibility that women might have been treated during early pregnancy. - If there is no mention in the abstract that the paper presents estimates for individual AEDs, the paper will not progress to level 2 screening. - Unadjusted results from randomized clinical trials are eligible for inclusion. - If an abstract suggests that comparisons are based only on mean values (e.g., “the mean birth weight among the exposed was lower than the mean birth weight among the unexposed”) and provides no hints that measures of association are presented in the full text, the paper is not eligible to progress to level 2 screening. |
| Refinement during level 2 screening |
| - Restriction to subjects on monotherapy with AEDs or with epilepsy is not enough adjustment to make a result eligible. |

AED = antiepileptic drug.

1. Eligibility criteria for results within eligible studies

| Result eligibility criteria |
| --- |
| For data extraction |
| - Each study can contribute to the study with up to one estimate per exposure/endpoint association in a given population (e.g., only one estimate of the effect of topiramate on head circumference in women with epilepsy compared to women with untreated epilepsy from Swedish data from years 2000-2005 will be extracted). If more than one is available, the decision on which estimate to extract will be based on relevance to the present literature search in terms of recency (more recent preferred), study size (larger preferred), and functional form of endpoints (continuous preferred over dichotomized forms). Standard outcome definitions will be preferred over study-specific outcome definitions (e.g., small for gestational age [SGA] defined with a threshold of p10 will be preferred over SGA defined over a threshold of p2.5). - If multiple estimates are available for the same exposure-endpoint association, including, for example, all women or only women with epilepsy, or more than one reference group, decisions on which one(s) to extract or use will depend on availability of data in other studies. - If results on the same association for one given period and for parts of the period are available (e.g., for 1995-2005 and for 1995-1997, 1998-2001, and 2002-2005), the overall estimate will be preferred. |
| For inclusion in analysis/dissemination |
| - When point estimates based on more than one form of adjustment on the same data (e.g., multivariate- and propensity score–adjusted odds ratios), all eligible results will be extracted, but one set will be kept for each analysis. Selection will be based on similarity to other studies in the comparison. |

AED = antiepileptic drug; OR = odds ratio; p = percentile; SGA = small for gestational age

## Literature search strategy and terms

The literature search was performed in PubMed on 26 May 2016 and updated on 12 April 2018; search terms are shown in Table 3. Reference lists of selected papers were reviewed to identify additional relevant publications.

1. PubMed search terms for systematic literature search

| # | Terms for | Terms |
| --- | --- | --- |
| 1 | AEDs overall  Individual drugs, including all in ATC | "Anticonvulsants"[Mesh] OR "Anticonvulsants" [Pharmacological Action] OR "Antiepileptic drugs"[All fields] OR Anticonvulsants[All fields] OR antiepileptic[All fields]  OR Acetazolamide[All fields] OR Alprazolam[All fields] OR Aminobutyric[All fields] OR Barbexaclone[All fields] OR Beclamide[All fields] OR Brivaracetam[All fields] OR Carbamazepine[All fields] OR Carisbamate[All fields] OR Clobazam[All fields] OR Clonazepam[All fields] OR Diazepam[All fields] OR Eslicarbazepine [All fields] OR Ethadione[All fields] OR Ethosuximide[All fields] OR Ethosuximide[All fields] OR Ethotoin[All fields] OR Felbamate[All fields] OR Gabapentin[All fields] OR Lacosamide[All fields] OR Lamotrigine[All fields] OR Levetiracetam[All fields] OR Lorazepam[All fields] OR Mephenytoin[All fields] OR Mesuximide[All fields] OR Metharbital[All fields] OR Methsuximide[All fields] OR Methylphenobarbital[All fields] OR Nitrazepam[All fields] OR Oxazepam[All fields] OR Oxcarbazepine[All fields] OR Paramethadione[All fields] OR Perampanel[All fields] OR Phenacemide[All fields] OR Pheneturide[All fields] OR Phenobarbital[All fields] OR Phensuximide[All fields] OR Phenytoin[All fields] OR Piracetam[All fields] OR Pregabalin[All fields] OR Primidone[All fields] OR Progabide[All fields] OR Retigabine[All fields] OR Rufinamide[All fields] OR Stiripentol[All fields] OR Sultiame[All fields] OR Tiagabine[All fields] OR Topiramate[All fields] OR Trimethadione[All fields] OR Trimethadone[All fields] OR Valeric[All fields] OR Valproic[All fields] OR Valproate[All fields] OR Valpromide[All fields] OR Vigabatrin[All fields] OR Zonisamide[All fields] |
| 2 | Birth weight  Preterm delivery, gestational age  Intrauterine growth restriction  Head circumference  Length at birth  Size at birth  Various pregnancy and fetal outcomes | "Birth Weight"[Mesh] OR "Infant, Extremely Low Birth Weight"[Mesh] OR "Infant, Very Low Birth Weight"[Mesh] OR "Infant, Low Birth Weight"[Mesh] OR "Infant, Small for Gestational Age"[Mesh]  OR  "Infant, Extremely Premature"[Mesh] OR "Premature Birth"[Mesh] OR "Infant, Premature"[Mesh] OR "gestational age at birth"[All fields] OR "Gestational Age"[Mesh] OR preterm[All fields] OR premature[All fields]  OR  "Fetal Growth Retardation"[Mesh] OR "Fetal Growth Retardation"[All fields] OR IUGR[All fields] OR "intrauterine growth retardation"[All fields] OR "Fetal Growth Restriction"[All fields] OR IUGR[All fields] OR "intrauterine growth restriction"[All fields]  OR  ((head OR cephalic) AND (circumference or perimeter) ) OR "Cephalometry"[Mesh]  OR  "length at birth"[All fields]  OR  "size at birth"[All fields]  OR  "pregnancy outcomes"[All fields] OR "perinatal outcomes"[All fields] OR "fetal outcomes"[All fields] OR "neonatal outcomes"[All fields] OR "pregnancy outcome"[All fields] OR "perinatal outcome"[All fields] OR "fetal outcome"[All fields] OR "neonatal outcome"[All fields] OR "reproductive outcomes"[All fields] OR "reproductive outcome"[All fields] OR "foetal outcome"[All fields] OR "foetal outcomes"[All fields] |
| 3 | Epidemiology, burden, safety | epidemiology[All fields] OR pharmacoepidemiology[All fields] OR safety[All fields] OR prevalence[All fields] OR incidence[All fields] OR burden[All fields] OR "Epidemiologic Methods"[Mesh:NoExp] OR “Epidemiologic Research Design"[Mesh] OR "Epidemiologic Studies"[Mesh] OR epidemiologic[All fields] OR epidemiological[All fields] |
| 4 | No case reports or papers on magnesium sulfate | "case reports"[Publication type] OR "case report"[title] OR "magnesium sulfate"[title] OR "magnesium sulphate"[title] |
| Search terms were combined as (#1 AND #2 AND #3 NOT #4) | | |

AED = antiepileptic drug; ATC = Anatomic Therapeutic Classification.

## Screening process, selection of studies and data extraction

- Level 1: review of titles or titles and abstracts, as needed, by one researcher (AVM)
- Level 2: review of full-text articles by one researcher (AVM)
- Data were extracted by one researcher (AVM). When numerical values were not directly available, they were either read from figures using the freely available app WebPlotDigitizer (<https://automeris.io/WebPlotDigitizer/>) or were provided by an author.

No formal quality assessment was conducted. Key extracted data were confirmed by a second researcher.

## Statistical Analysis

Meta-analysis was considered depending on the suitability of the extracted results. At least three independent data points with reasonably similar specifications (e.g., exposure, monotherapy or polytherapy, reference group, statistical model, adjustment) were required.

# Results

## Literature Search and Screening Process

The literature search, conducted on 26 May 2016, retrieved 872 PubMed entries published from 1962 to 2016 (Figure 1). Seventy-one papers were examined in full (level 2 screening). Ten papers were selected for inclusion in this systematic search. From the reference lists of these 10 papers, 15 additional papers were evaluated, published from 1976 to 2012. Six papers progressed to level 2 screening. One paper was selected for this systematic review for a total of 11 included papers. The literature search was updated on 12 April 2018 using the same search terms and restricting the search to entries with a publication date of 26 May 2016 or later. This search retrieved 59 papers published in 2016 to 2018, of which 10 were examined in full and 4 were selected for inclusion. The review of reference lists did not identify additional papers for review. A total of 15 papers were considered eligible.

1.
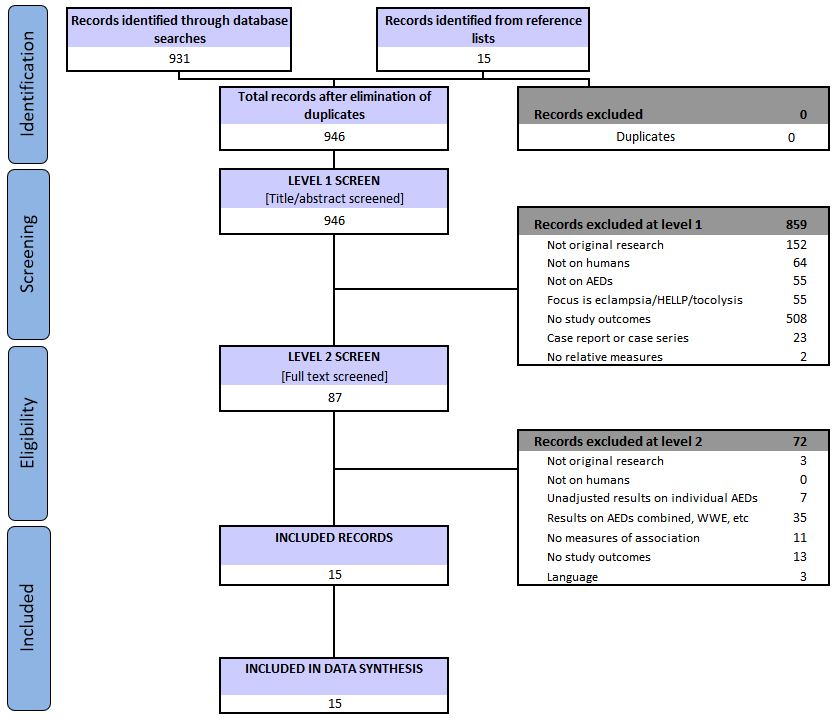
PRISMA Flow Diagram

AED = antiepileptic drug; HELLP = hemolysis elevated liver enzymes and low platelet count syndrome; WWE = women with epilepsy.

## Extracted Data

The 15 included papers were published between 1981 and 2017 in English.[^3-17^](#_ENREF_3) Two papers had partially overlapping study populations (from nationwide Swedish registries),[^3^](#_ENREF_3)^,^[^16^](#_ENREF_16) and in another two, one population was a subset of the other.[^13^](#_ENREF_13)^,^[^14^](#_ENREF_14) We extracted 244 unique results. Some results represented minor variants of others (e.g., multivariable- and propensity score–adjusted results from one paper); some results were subsets of others (e.g., one association was measured in the entire study population and in a subset of nonsmokers).

None of the papers presented results in the form of z-scores, which we preferred because z-scores enable assessing size independently of any effect on pregnancy duration. The studies were published between 1981 and 2017; 9 used data from the Nordic countries,[^3^](#_ENREF_3)^,^[^4^](#_ENREF_4)^,^[^8-11^](#_ENREF_8)^,^[^13^](#_ENREF_13)^,^[^14^](#_ENREF_14)^,^[^16^](#_ENREF_16) 2 of which were from Sweden,[^3^](#_ENREF_3)^,^[^16^](#_ENREF_16) reflecting the excellent quality of register data from these countries. These two studies use data that overlap with our study; but statistical analyses, including the reference for exposure, are different from ours.

Eleven papers reported on carbamazepine as the exposure drug (i.e., not as reference group),[^3^](#_ENREF_3)^,^[^4^](#_ENREF_4)^,^[^6^](#_ENREF_6)^,^[^8-14^](#_ENREF_8)^,^[^16^](#_ENREF_16) providing results on monotherapy (8 papers), polytherapy (3 papers), or either (5 papers). Additionally, 11 papers reported on valproic acid (7 on monotherapy, 2 on polytherapy, 6 on either),[^3-6^](#_ENREF_3)^,^[^9-15^](#_ENREF_9) 6 on lamotrigine (3 on monotherapy, 2 on polytherapy and 5 on either),[^3^](#_ENREF_3)^,^[^4^](#_ENREF_4)^,^[^10^](#_ENREF_10)^,^[^11^](#_ENREF_11)^,^[^13^](#_ENREF_13)^,^[^14^](#_ENREF_14) 4 on levetiracetam (2 papers on monotherapy, 1 on polytherapy, 3 on either),[^4^](#_ENREF_4)^,^[^6^](#_ENREF_6)^,^[^10^](#_ENREF_10)^,^[^14^](#_ENREF_14) and 1 on pregabalin (either monotherapy or polytherapy).[^17^](#_ENREF_17)

Most commonly (12 papers),[^3^](#_ENREF_3)^,^[^4^](#_ENREF_4)^,^[^7-11^](#_ENREF_7)^,^[^13-17^](#_ENREF_13) the reference was unexposed women (no AEDs and no maternal epilepsy, no AEDs, the general population, or no AEDs, maternal epilepsy, or other chronic diseases). Other reference groups were lamotrigine (3 papers),[^6^](#_ENREF_6)^,^[^7^](#_ENREF_7)^,^[^12^](#_ENREF_12) carbamazepine (2 papers),[^5^](#_ENREF_5)^,^[^12^](#_ENREF_12) and phenytoin (1 paper).[^12^](#_ENREF_12)

The most common endpoint was birth weight (12 papers),[^4^](#_ENREF_4)^,^[^6-16^](#_ENREF_6) implemented in 7 forms: continuous, 2 forms of low birth weight (less than 2,500 g, and between 1,500 and 2,499 g), 3 forms of SGA (less than 2 SDs, less than p10, and less than p2.5), and 2 forms of large for gestational age (more than 2 SDs, and more than p10). Head circumference was reported in 7 papers,[^3^](#_ENREF_3)^,^[^5^](#_ENREF_5)^,^[^8^](#_ENREF_8)^,^[^10^](#_ENREF_10)^,^[^11^](#_ENREF_11)^,^[^14^](#_ENREF_14)^,^[^16^](#_ENREF_16) and was implemented as a continuous variable and as 4 different dichotomous definitions (microcephaly): less than 32 cm, less than 2 SDs, less than p10, and less than p2.5). Duration of pregnancy (or gestational age at birth) was reported in 5 papers[^4^](#_ENREF_4)^,^[^10^](#_ENREF_10)^,^[^13^](#_ENREF_13)^,^[^16^](#_ENREF_16)^,^[^17^](#_ENREF_17) in 2 functional forms: continuous and preterm (less than 37 weeks). Birth length was reported in 2 papers,[^8^](#_ENREF_8)^,^[^16^](#_ENREF_16) in 2 forms: continuous and binarized as less than 47 cm. No results presented outcomes as z-scores, although 1 paper presented a custom-designed SD score.[^3^](#_ENREF_3)

Of the three papers used lamotrigine as the reference group,[^6^](#_ENREF_6)^,^[^7^](#_ENREF_7)^,^[^12^](#_ENREF_12) 2 were derived from the same data source.[^6^](#_ENREF_6)^,^[^7^](#_ENREF_7) The three papers reported at individual-drug level on SGA only. We did not combine these results.

Other results are presented in the body of the paper.

# References

1. Margulis AV, Oberg AS, Hernandez-Diaz S. Antiepileptic drugs in pregnancy: searching for a reference drug for comparative safety. Pharmacoepidemiol Drug Saf. 2017;26(Suppl 2):408-9.

2. Viale L, Allotey J, Cheong-See F, Arroyo-Manzano D, McCorry D, Bagary M, et al. Epilepsy in pregnancy and reproductive outcomes: a systematic review and meta-analysis. Lancet. 2015 Nov 7;386(10006):1845-52.

3. Almgren M, Kallen B, Lavebratt C. Population-based study of antiepileptic drug exposure in utero--influence on head circumference in newborns. Seizure. 2009 Dec;18(10):672-5.

4. Artama M, Gissler M, Malm H, Ritvanen A. Effects of maternal epilepsy and antiepileptic drug use during pregnancy on perinatal health in offspring: nationwide, retrospective cohort study in Finland. Drug Saf. 2013 May;36(5):359-69.

5. Battino D, Kaneko S, Andermann E, Avanzini G, Canevini MP, Canger R, et al. Intrauterine growth in the offspring of epileptic women: a prospective multicenter study. Epilepsy Res. 1999 Aug;36(1):53-60.

6. Hernandez-Diaz S, McElrath TF, Pennell PB, Hauser WA, Yerby M, Holmes LB. Fetal growth and premature delivery in pregnant women on antiepileptic drugs. Ann Neurol. 2017 Sep;82(3):457-65.

7. Hernandez-Diaz S, Mittendorf R, Smith CR, Hauser WA, Yerby M, Holmes LB, et al. Association between topiramate and zonisamide use during pregnancy and low birth weight. Obstet Gynecol. 2014 Jan;123(1):21-8.

8. Hiilesmaa VK, Teramo K, Granstrom ML, Bardy AH. Fetal head growth retardation associated with maternal antiepileptic drugs. Lancet. 1981 Jul 25;2(8239):165-7.

9. Hvas CL, Henriksen TB, Ostergaard JR, Dam M. Epilepsy and pregnancy: effect of antiepileptic drugs and lifestyle on birthweight. BJOG. 2000 Jul;107(7):896-902.

10. Kilic D, Pedersen H, Kjaersgaard MI, Parner ET, Vestergaard M, Sorensen MJ, et al. Birth outcomes after prenatal exposure to antiepileptic drugs--a population-based study. Epilepsia. 2014 Nov;55(11):1714-21.

11. Kolstad E, Veiby G, Gilhus NE, Bjork M. Overweight in epilepsy as a risk factor for pregnancy and delivery complications. Epilepsia. 2016 Nov;57(11):1849-57.

12. Pennell PB, Klein AM, Browning N, Baker GA, Clayton-Smith J, Kalayjian LA, et al. Differential effects of antiepileptic drugs on neonatal outcomes. Epilepsy Behav. 2012 Aug;24(4):449-56.

13. Veiby G, Daltveit AK, Engelsen BA, Gilhus NE. Pregnancy, delivery, and outcome for the child in maternal epilepsy. Epilepsia. 2009 Sep;50(9):2130-9.

14. Veiby G, Daltveit AK, Engelsen BA, Gilhus NE. Fetal growth restriction and birth defects with newer and older antiepileptic drugs during pregnancy. J Neurol. 2014 Mar;261(3):579-88.

15. Wen X, Hartzema A, Delaney JA, Brumback B, Liu X, Egerman R, et al. Combining adverse pregnancy and perinatal outcomes for women exposed to antiepileptic drugs during pregnancy, using a latent trait model. BMC Pregnancy Childbirth. 2017 Jan 6;17(1):10.

16. Wide K, Winbladh B, Tomson T, Kallen B. Body dimensions of infants exposed to antiepileptic drugs in utero: observations spanning 25 years. Epilepsia. 2000 Jul;41(7):854-61.

17. Winterfeld U, Merlob P, Baud D, Rousson V, Panchaud A, Rothuizen LE, et al. Pregnancy outcome following maternal exposure to pregabalin may call for concern. Neurology. 2016 Jun 14;86(24):2251-7.
