# Supplementary material for: Relation of in-utero exposure to antiepileptic drugs to pregnancy duration and size at birth
Source: PLoS One. 2019 Aug 5;14(8):e0214180. doi: 10.1371/journal.pone.0214180 (PMC6681941; doi:10.1371/journal.pone.0214180)
Supplement: S2 File — (DOCX) [file pone.0214180.s002.docx]

**Variable definitions**

This is supporting information to:

Relation of in-utero exposure to antiepileptic drugs to pregnancy duration and size at birth

Margulis AV, Hernandez-Diaz H, McElrath T, Rothman KJ, Plana E, Almqvist C, D´Onofrio BM, Oberg AS

**Table. Characteristics of Study Population and Other Variables: Definitions**

| Cohort Characteristic | Time Frame | Functional Form of Variable and Comments | Registers Used | Non-ICD– or ATC-Coded Variables | Codes | | | |
| --- | --- | --- | --- | --- | --- | --- | --- | --- |
|  |  |  |  |  | ATC | ICD-8 | ICD-9 | ICD-10 |
| First day of last menstrual period | n/a | Date.  Estimated as date of birth minus gestational age at birth. | MBR | Yes | n/a | n/a | n/a | n/a |
| Year | At delivery | Categorical: approximate quintiles, 1996-2001, 2002-2006, 2007-2009, 2010-2011, 2012-2013. | MBR | Yes | n/a | n/a | n/a | n/a |
| Maternal age | At delivery | Categorical: <25, 25-29, 30-34, ≥35 years. | MBR | Yes | n/a | n/a | n/a | n/a |
| Maternal country of origin | As recorded in prenatal care | Categorical: Nordic countries, other European countries, Asia, others. | MBR | Yes | n/a | n/a | n/a | n/a |
| Maternal education | At delivery | Categorical: up to 12 years, ≥13 years. | Swedish Register of Education | Yes | n/a | n/a | n/a | n/a |
| Maternal marital status | As recorded in prenatal care | Categorical: lives with child’s father, does not live with child’s father. | MBR | Yes | n/a | n/a | n/a | n/a |
| Maternal early pregnancy BMI | At prenatal care registration | Categorical: BMI (kg/m^2^) <18.5, 18.5 - <25, 25 - <30; ≥30. Categories taken from CDC classification (<https://www.cdc.gov/obesity/adult/defining.html>, accessed 3 November 2017) and Derraik JGB et al. Obesity rates in two generations of Swedish women entering pregnancy, and associated obesity risk among adult daughters. Scientific Reports. 2015;5:16692. doi:10.1038/srep16692.  Early pregnancy BMI was calculated from weight and height recorded at the MBR at the first prenatal visit or, in 13 women without such information, from coded entries for obesity in their medical records in the 3 years before pregnancy. There were no recorded ICD codes for other BMI categories in the study population. | MBR | Yes | n/a | n/a | n/a | n/a |
|  | 3 years before LMP (LMP not included) | BMI (kg/m^2^) ≥30.  BMI from codes to supplement data from the MBR. | MBR  National Patient Register | n/a | n/a | 277 | V85.3 V85.4 | E66 |
|  | 3 years before LMP (LMP not included) | BMI: 25 to <30. | MBR  National Patient Register | n/a | n/a | n/a | V85.2 | n/a |
|  | 3 years before LMP (LMP not included) | BMI: 18.5 to <25. | MBR  National Patient Register | n/a | n/a | n/a | V85.1 | n/a |
|  | 3 years before LMP (LMP not included) | BMI <18.5. | MBR  National Patient Register | n/a | n/a | n/a | V85.0 | n/a |
| Smoking | Any time in pregnancy | Categorical: nonsmoker, smoker.  The MBR contains information on smoking in the 3 months before pregnancy, smoking at prenatal care registration, and smoking in weeks 30-32. In this study, women were considered smokers in a given pregnancy if they reported smoking at prenatal care registration or in weeks 30-32 or if they reported smoking in the 3 months before pregnancy and values for the other two time points were missing. | MBR | Yes | n/a | n/a | n/a | n/a |
| Alcohol dependence | All history up to the end of this pregnancy | Categorical: Y/N.  Also includes alcohol-induced mental disorders. | National Patient Register  MBR | n/a | n/a | 291 303 | 291 303 | F10 |
| Epilepsy | All history up to the end of this pregnancy | Categorical: Y/N.  ICD-10 codes from Bolin K et al. Regional variation in prevalence and healthcare utilization due to epilepsy in Sweden. Acta Neurologica. 2014;130(6):354-9. | MBR National Patient Register | Yes | n/a | 345 | 345 649E | G40 G41 |
| Depression | 5 years before end of this pregnancy | Categorical: Y/N.  ICD-10 from case definition four in Fiest et al. Systematic review and assessment of validated case definitions for depression in administrative data. BMC Psychiatry 2014, 14:289. ICD-10 F41.2 is anxiety with depression and is included in both depression and other psychiatric disorders. | MBR  National Patient Register | n/a | n/a | n/a | 300E 296C 296D 311 | F32 F33.0 F33.1 F33.2 F33.3 F33.8 F33.9 F34.1 F41.2 |
| Bipolar disorder | All history up to the end of this pregnancy | Categorical: Y/N.  ICD-10 F31 from Crump C et al. Comorbidities and Mortality in Bipolar Disorder A Swedish National Cohort Study. JAMA Psychiatry. 2013;70(9):931-939. doi: 10.1001/jamapsychiatry.2013.1394. | MBR  National Patient Register | n/a | N05AN01 | n/a | 296A 296E 296F 296H 296W | F31 |
| Other psychiatric disorders | All history up to the end of this pregnancy | Categorical: Y/N.  Disorders included are schizophrenia and related psychoses, mania, phobic disorders, panic attacks, obsessive-compulsive disorders, eating disorders.  Chlorpromazine, commonly used as an antiemetic in pregnancy, is not included (ATC code N05AA01); lithium is also not included (ATC code N05AN01) to decrease overlap with variable for bipolar disorder. | MBR  National Patient Register  Prescribed Drug Register | n/a | N05A | 295 297 300 | 295 297 298 296B 783A 307F 300A 300B 300C 300D | F2 F30 F50 F40 F41 F42 |
| Migraine | All history up to the end of this pregnancy | Categorical: Y/N.  Cluster headaches and others are not included (G44).  ATC codes in N02C (antimigraine preparations) plus drugs with level A of evidence in Loder E et al. The 2012 AHS/AAN guidelines for prevention of episodic migraine: a summary and comparison with other recent clinical practice guidelines. Headache. 2012;52(6):930-45. doi: 10.1111/j.1526-4610.2012.02185.x. (except AEDs): propranolol, timolol (systemic) and metoprolol. | MBR  National Patient Register  Prescribed Drug Register | n/a | N02C C07AA05 C07AA06 C07AB02 | 346 | 346 | G43 |
| Chronic pain or use of opioids in the last 5 years | Diagnoses: 5 years before end of this pregnancy. Dispensings: 5 years before end of this pregnancy to 30 days before end of this pregnancy to avoid capturing prescriptions intended for postpartum use | Categorical: Y/N.  Conditions included are fibromyalgia, generalized pain, central neuropathic pain, postherpetic neuralgia, and related diagnoses, sciatica and related pain syndromes.  Conditions identified from Wiffen PJ et al. Antiepileptic drugs for neuropathic pain and fibromyalgia - an overview of Cochrane reviews. Cochrane Database of Systematic Reviews 2013, Issue 11. DOI: 10.1002/14651858.CD010567.pub2 and from Wettermark B et al. Pregabalin is increasingly prescribed for neuropathic pain, generalised anxiety disorder and epilepsy but many patients discontinue treatment. International Journal of Clinical Practice. 2014;68:104-110. In general, codes for pain were retained rather than codes for conditions that have pain as a symptom. Added ICD-10 F45.4. | MBR  National Patient Register  Prescribed Drug Register | n/a | N02A | 351 | 338A 338C 338X 350B 729B 729C 729F 350C 354A 355H 354E 355B 723C 723D 723B 724D 724C | M79.0 M79.1 M79.2 M79.6 M79.7 R52.1 R52.2 R52.9 G35.9 G50.0 G50.1 G53.0 G56.0 G56.4 G57.1 M53.0 M53.1 F45.4 M54 |
| Restless legs syndrome | All history up to the end of this pregnancy | Categorical: Y/N.  The specific ICD-9 code is 333.94. Because other elements in 333.9 seem infrequent, 333.9 was used for restless legs syndrome. Similarly, ICD-10 G25.8 also includes other movement disorders but was included here.  Drugs from level-A evidence in Winkelman JW. Practice guideline summary: Treatment of restless legs syndrome in adults. Neurology 2016 13;87(24):2585-2593. Doi: [http:/​/​dx.​doi.​org/​10.​1212/​WNL.​0000000000003388](http://dx.doi.org/10.1212/WNL.0000000000003388) (except gabapentin): pramipexole (ATC code N04BC05), rotigotine (N04BC09), cabergoline (N04BC06; G02CB03 was not retained as this lower-dose form is used to suppress lactation and for other gynecological uses). Used for descriptive purposes. | MBR  National Patient Register  Prescribed Drug Register | n/a | N04BC05 N04BC09 N04BC06 | n/a | 333X | G25.8 |
| Diabetes (preexisting and gestational combined) | All history up to the end of this pregnancy | Categorical: Y/N.  Preexisting diabetes. | MBR  National Patient Register | Yes | n/a | 250 | 250 648A | E10 E11 E12 E13 E14 O24.0 O24.1 O24.2 O24.3 |
|  | Index pregnancy (LMP included) | Categorical: Y/N.  Gestational diabetes. |  | Yes | n/a | n/a | 648W | O24.4 O24.9 |
| Hypertension (preexisting and gestational combined) | All history up to the end of this pregnancy | Categorical: Y/N.  Preexisting hypertension. | MBR  National Patient Register | n/a | n/a | 400 401 402 403 404 | 401 402 403 404 405 642A 642B 642C | I10 I11 I12 I13 I14 I15 O10 O11 O12 |
|  | Index pregnancy (LMP included) | Categorical: Y/N.  Gestational hypertension. | MBR  National Patient Register | n/a | n/a | 637.01 | 642D 642X | O13 O16 |
| AED polytherapy in this pregnancy | In index pregnancy | Categorical: Y/N.  More than one dispensing in time window. | MBR  Prescribed Drug Register | n/a | N03 | n/a | n/a | n/a |
| Antidepressants in this pregnancy | In index pregnancy | Categorical: Y/N. | MBR  Prescribed Drug Register | n/a | N06A | n/a | n/a | n/a |
| SSRIs this pregnancy | In index pregnancy | Categorical: Y/N. | MBR  Prescribed Drug Register | n/a | N06AB | n/a | n/a | n/a |
| Non-SSRI antidepressants this pregnancy | In index pregnancy | Categorical: Y/N.  For adjustment in sensitivity analyses in which SSRI use is examined as an effect modifier | MBR  Prescribed Drug Register | n/a | N06A except N06AB | n/a | n/a | n/a |
| Antipsychotics in this pregnancy | In index pregnancy | Categorical: Y/N.  Excluded: chlorpromazine (ATC code N05AA01) and lithium (ATC code N05AN01). See comment in variable “other psychiatric disorders.” | MBR  Prescribed Drug Register | n/a | N05A | n/a | n/a | n/a |
| Migraine Treatment in this pregnancy | In index Pregnancy | Categorical: Y/N.  See comment in variable “other psychiatric disorders.” | MBR  Prescribed Drug Register | n/a | N02C C07AA05 C07AA06 C07AB02 | n/a | n/a | n/a |
| Opioids in this pregnancy | Index pregnancy - until 30 days before the end of this pregnancy to avoid capturing prescriptions intended for postpartum use | Categorical: Y/N. | MBR  Prescribed Drug Register | n/a | N02A | n/a | n/a | n/a |
| Sex of offspring | n/a | Female or male. | MBR | Yes | n/a | n/a | n/a | n/a |
| Major congenital malformations (offspring) | First year of life | Categorical: Y/N. | MBR National Patient Register | Yes | n/a | n/a | 74 75 | Q |
|  |  | Entries to exclude minor malformation from among codes for malformations.  List from European Surveillance of Congenital Anomalies (EUROCAT). Minor anomalies for exclusion (used until 2004). Available at <http://www.eurocat-network.eu/content/EUROCAT-Old-List-Minor-Anomalies.pdf>, accessed 16 September 2017. This list was used instead of the most current one because this one includes ICD-9 and -10 codes, while the new one only includes ICD-10 codes. Handling of ICD-9 codes for minor malformations: only 4-digit codes starting in 74 and 75 were retained; the fourth digit was modified per Swedish implementation. ICD-10 code handling: only codes starting in Q were retained. | MBR National Patient Register | n/a | n/a | n/a | 744D 747F 747A 750A 752F 750G 757B 757W | Q10.5 Q17.9 Q27.0 Q25.0 Q38.1 Q53 Q540 Q66.8 Q66.2 Q66.4 Q76.0 Q67.6 Q76.7 Q30.9 Q18.9 Q83.3 Q40.1 Q82.80 Q82.81 Q82.5 |
| Chromosomal anomalies (offspring) | All available information | Categorical: Y/N.  This is an exclusion criterion. | MBR National Patient Register | n/a | n/a | 759.3 759.4 759.5 759.6 759.7 759.8 759.9 | 758 | Q9 |

AAN = American Academy of Neurology; AED = antiepileptic drug; AHS = American Headache Society; ATC = Anatomical Therapeutic Chemical; BMI = body mass index; CDC = Centers of Disease Control and Prevention; ICD = International Classification of Diseases; ICD-8 = ICD-Eighth Revision; ICD-9 = ICD-Ninth Revision; ICD-10 = ICD-Tenth Revision; LMP = first day of last menstrual period; MBR = Medical Birth Register; n/a = not applicable; SSRI = selective serotonin reuptake inhibitor; Y/N = yes/no.

Note: ATC codes were retrieved from self-reports and dispensed prescriptions. For variables based on diagnoses and prescriptions, the delivery day was not included. Included in the variable definition are all codes starting in the codes listed in this table; e.g., the ICD-10 definition of chromosomal abnormalities included all codes starting in Q9. Variables were defined by the presence of at least one record with an appropriate code in the stated time window. Information in the Swedish National Patient Register is coded in ICD-8, -9 and -10, Swedish implementation. ICD-8 codes were extracted from <http://www.wolfbane.com/icd/icd8.htm>. ICD-9 codes were extracted from <http://icd9.chrisendres.com/index.php?action=child&recordid=6306>. ICD-10 codes were extracted from <http://apps.who.int/classifications/icd10/browse/2015/en#/C80>. For the three coding systems, some variables were developed using previously developed code lists from publications and the research team. Also, documents describing the Swedish implementation of ICD coding were consulted.
