# Supplementary material for: Relation of in-utero exposure to antiepileptic drugs to pregnancy duration and size at birth
Source: PLoS One. 2019 Aug 5;14(8):e0214180. doi: 10.1371/journal.pone.0214180 (PMC6681941; doi:10.1371/journal.pone.0214180)
Supplement: S3 File — (DOCX) [file pone.0214180.s003.docx]

Supplemental Result Tables

This is supporting information to:

Relation of in-utero exposure to antiepileptic drugs to pregnancy duration and size at birth

Margulis AV, Hernandez-Diaz H, McElrath T, Rothman KJ, Plana E, Almqvist C, D´Onofrio BM, Oberg AS

list of Tables

[Table A. Means and percentages of study outcomes in infants exposed to the individual study drugs 2](#_Toc14109638)

[Table B. Association between in-utero carbamazepine exposure and the endpoints duration of pregnancy and size at birth 3](#_Toc14109639)

[Table C. Effect-measure modification analysis for the association between in-utero carbamazepine exposure and the endpoints duration of pregnancy and size at birth 10](#_Toc14109640)

[Table D. Association between in-utero pregabalin exposure and the endpoints duration of pregnancy and size at birth 11](#_Toc14109641)

[Table E. Effect-measure modification analysis for the association between in-utero pregabalin exposure and the endpoints duration of pregnancy and size at birth 18](#_Toc14109642)

[Table F. Association between in-utero levetiracetam exposure and the endpoints duration of pregnancy and size at birth 19](#_Toc14109643)

[Table G. Effect-measure modification analysis for the association between in-utero levetiracetam exposure and the endpoints duration of pregnancy and size at birth 26](#_Toc14109644)

[Table H. Association between in-utero valproic acid exposure and the endpoints duration of pregnancy and size at birth 27](#_Toc14109645)

[Table I. Effect-measure modification analysis for the association between in-utero valproic acid exposure and the endpoints duration of pregnancy and size at birth 34](#_Toc14109646)

[Table J. Association between in-utero lamotrigine exposure and the endpoints duration of pregnancy and size at birth 35](#_Toc14109647)

[Table K. Estimated effects from linear regression models for key variables 37](#_Toc14109648)

1. Means and percentages of study outcomes in infants exposed to the individual study drugs

|  | **Duration of pregnancy (days)** | | | **Preterm**  **delivery** | | | **Birth weight**  **z-score** | | | **Small for**  **gestational age** | | | **Birth length**  **z-score** | | | **Head circumference**  **z-score** | | | |  |
| --- | --- | --- | --- | --- | --- | --- | --- | --- | --- | --- | --- | --- | --- | --- | --- | --- | --- | --- | --- | --- |
|  | **Infants, n** | **Mean** | **SD** | **Infants, n** | **Case infants, n** | **%** | **Infants, n** | **Mean** | **SD** | **Infants, n** | **Infants, n** | **%** | **Infants, n** | **Mean** | **SD** | | **Infants, n** | **Mean** | **SD** | |
| Carbamazepine | 2,095 | 275.8 | 15.4 | 2,095 | 171 | 8.2 | 2,089 | -0.1 | 1.1 | 2,047 | 78 | 3.8 | 2,062 | -0.1 | 1.0 | | 1,980 | -0.0 | 1.1 | |
| Pregabalin | 562 | 273.0 | 15.4 | 562 | 76 | 13.5 | 561 | -0.2 | 1.1 | 547 | 22 | 4.0 | 553 | -0.3 | 1.0 | | 549 | 0.1 | 1.1 | |
| Levetiracetam | 307 | 275.1 | 17.5 | 307 | 29 | 9.4 | 306 | -0.1 | 1.0 | 298 | 8 | 2.7 | 303 | -0.0 | 1.0 | | 296 | 0.1 | 1.1 | |
| Valproic acid | 1,137 | 276.5 | 18.2 | 1,137 | 117 | 10.3 | 1,130 | -0.0 | 1.2 | 1,100 | 52 | 4.7 | 1,101 | 0.1 | 1.1 | | 1,065 | 0.0 | 1.1 | |
| Lamotrigine | 2,254 | 276.5 | 14.7 | 2,254 | 180 | 8.0 | 2,248 | 0.0 | 1.1 | 2,184 | 52 | 2.4 | 2,218 | -0.0 | 1.0 | | 2,193 | 0.2 | 1.1 | |

|  | **Microcephaly** | | | **Birth weight (grams)** | | | **Birth length (cm)** | | | **Head circumference (cm)** | | |
| --- | --- | --- | --- | --- | --- | --- | --- | --- | --- | --- | --- | --- |
|  | **Infants, n** | **Case infants, n** | **%** | **Infants, n** | **Mean** | **SD** | **Infants, n** | **Mean** | **SD** | **Infants, n** | **Mean** | **SD** |
| Carbamazepine | 1,980 | 60 | 3.0 | 2,089 | 3,436 | 634 | 2,062 | 49.9 | 2.8 | 1,980 | 34.5 | 1.9 |
| Pregabalin | 549 | 15 | 2.7 | 561 | 3,311 | 610 | 553 | 49.2 | 2.7 | 549 | 34.6 | 1.9 |
| Levetiracetam | 296 | 9 | 3.0 | 306 | 3,401 | 618 | 303 | 49.9 | 3.0 | 296 | 34.6 | 2.1 |
| Valproic acid | 1,065 | 41 | 3.8 | 1,130 | 3,452 | 685 | 1,101 | 50.2 | 3.3 | 1,065 | 34.7 | 1.9 |
| Lamotrigine | 2,193 | 48 | 2.2 | 2,248 | 3,471 | 601 | 2,218 | 50.0 | 2.6 | 2,193 | 34.8 | 1.8 |

SD = standard deviation.

1. Association between in-utero carbamazepine exposure and the endpoints duration of pregnancy and size at birth

|  | | **Difference (95% CI)** | | | | **Odds ratio** |
| --- | --- | --- | --- | --- | --- | --- |
|  | | | **At percentile** | | | **(95% CI)** |
|  | **Exposed to carbamazepine/ reference** | **Mean** | **10^th^** | **50^th^** | **90^th^** |  |
| **Pregnancy duration (days)                                               Preterm birth** | | | | | | |
| Use any time in pregnancy, carbamazepine vs. lamotrigine | 1,975 / 2,123 | -1.3 (-2.3 to -0.3) | -1.1 (-3.1 to 0.9) | -0.9 (-1.8 to 0.1) | -0.1 (-1.3 to 1.0) | 1.2 (0.9 to 1.5) |
| Use in first trimester, carbamazepine vs. lamotrigine | 1,686 / 1,930 | -1.6 (-2.7 to -0.5) | -2.3 (-4.5 to -0.1) | -0.9 (-1.8 to 0.0) | -0.5 (-1.5 to 0.6) | 1.3 (1.0 to 1.8) |
| Continuers, carbamazepine vs. lamotrigine | 459 / 1,013 | -1.3 (-3.0 to 0.3) | 0.0 (-3.8 to 3.8) | -0.3 (-2.0 to 1.3) | -0.5 (-1.9 to 0.9) | 1.1 (0.7 to 1.7) |
| Mother with epilepsy, carbamazepine vs. lamotrigine | 1,665 / 1,447 | -1.3 (-2.4 to -0.2) | -1.6 (-3.5 to 0.3) | -0.5 (-1.5 to 0.5) | -0.2 (-1.3 to 0.9) | 1.3 (0.9 to 1.7) |
| Mother with chronic pain, carbamazepine vs. lamotrigine | 259 / 541 | -1.5 (-4.2 to 1.1) | -4.5 (-10.5 to 1.5) | -0.7 (-2.7 to 1.4) | 0.1 (-2.7 to 2.8) | 1.3 (0.7 to 2.3) |
| Monotherapy, carbamazepine vs. lamotrigine | 1,808 / 1,787 | -1.3 (-2.4 to -0.2) | -1.0 (-3.2 to 1.3) | -0.6 (-1.6 to 0.4) | 0.0 (-1.1 to 1.1) | 1.2 (0.9 to 1.6) |
| Polytherapy, carbamazepine vs. lamotrigine | 167 / 336 | -2.4 (-5.8 to 1.0) | -6.1 (-15.1 to 2.8) | -2.0 (-5.4 to 1.4) | -1.5 (-4.0 to 1.0) | 1.7 (0.9 to 3.3) |
| Definite exposure, carbamazepine vs. lamotrigine | 535 / 1,072 | -1.5 (-3.0 to 0.1) | -2.1 (-5.5 to 1.3) | -0.1 (-1.5 to 1.2) | -0.3 (-1.8 to 1.1) | 1.4 (0.9 to 2.1) |
| Complete-case analysis, carbamazepine vs. lamotrigine | 1,720 / 1,909 | -1.1 (-2.2 to -0.0) | -1.1 (-3.3 to 1.1) | -0.9 (-2.0 to 0.1) | -0.3 (-1.3 to 0.7) | 1.0 (0.8 to 1.4) |
| Singletons with no MCMs, carbamazepine vs. lamotrigine | 1,798 / 1,916 | -1.3 (-2.3 to -0.2) | -1.5 (-3.4 to 0.4) | -1.0 (-1.8 to -0.2) | -0.2 (-1.2 to 0.8) | 1.2 (0.9 to 1.7) |
| First infant per woman, carbamazepine vs. lamotrigine | 1,450 / 1,624 | -1.2 (-2.5 to 0.1) | -0.5 (-3.0 to 2.1) | -0.5 (-1.6 to 0.5) | -0.5 (-1.7 to 0.7) | 1.1 (0.8 to 1.5) |
| Female infants, carbamazepine vs. lamotrigine | 926 / 1,118 | -0.9 (-2.3 to 0.6) | -1.8 (-4.7 to 1.0) | -0.8 (-1.9 to 0.4) | 0.1 (-1.2 to 1.5) | 1.1 (0.8 to 1.7) |
| Male infants, carbamazepine vs. lamotrigine | 1,049 / 1,005 | -1.7 (-3.3 to -0.2) | -0.6 (-3.3 to 2.1) | -0.8 (-2.1 to 0.5) | -0.3 (-1.8 to 1.1) | 1.2 (0.8 to 1.8) |
| High vs. low dose of carbamazepine | 264 / 275 | -4.6 (-7.5 to -1.6) | -6.8 (-12.6 to -0.9) | -3.4 (-5.8 to -0.9) | -2.1 (-4.7 to 0.4) | 2.8 (1.3 to 6.0) |
| **Birth weight z-score                                                            SGA** | | | | | | |
| Use any time in pregnancy, carbamazepine vs. lamotrigine | 1,988 / 2,147 | -0.1 (-0.2 to -0.0) | -0.0 (-0.1 to 0.1) | -0.1 (-0.2 to -0.0) | -0.2 (-0.3 to -0.1) | 1.4 (0.9 to 2.1) |
| Use in first trimester, carbamazepine vs. lamotrigine | 1,699 / 1,953 | -0.1 (-0.2 to -0.0) | -0.1 (-0.2 to 0.1) | -0.1 (-0.2 to -0.0) | -0.2 (-0.3 to -0.1) | 1.7 (1.0 to 2.6) |
| Continuers, carbamazepine vs. lamotrigine | 466 / 1,021 | -0.1 (-0.2 to -0.0) | -0.1 (-0.3 to 0.1) | -0.1 (-0.2 to 0.0) | -0.2 (-0.3 to -0.0) | 1.3 (0.7 to 2.6) |
| Mother with epilepsy, carbamazepine vs. lamotrigine | 1,676 / 1,459 | -0.1 (-0.2 to -0.0) | 0.0 (-0.1 to 0.1) | -0.1 (-0.2 to -0.1) | -0.2 (-0.3 to -0.0) | 1.2 (0.8 to 1.9) |
| Mother with chronic pain, carbamazepine vs. lamotrigine | 263 / 552 | -0.2 (-0.3 to 0.0) | -0.1 (-0.4 to 0.3) | -0.1 (-0.3 to 0.1) | -0.1 (-0.4 to 0.1) | 1.8 (0.8 to 4.2) |
| Monotherapy, carbamazepine vs. lamotrigine | 1,821 / 1,808 | -0.1 (-0.1 to 0.0) | 0.1 (-0.1 to 0.2) | -0.1 (-0.1 to 0.0) | -0.2 (-0.3 to -0.0) | 1.3 (0.8 to 2.0) |
| Polytherapy, carbamazepine vs. lamotrigine | 167 / 339 | -0.5 (-0.7 to -0.3) | -0.6 (-0.9 to -0.3) | -0.5 (-0.7 to -0.2) | -0.3 (-0.8 to 0.1) | 4.2 (1.2 to 14.4) |
| Definite exposure, carbamazepine vs. lamotrigine | 542 / 1,083 | -0.1 (-0.2 to -0.0) | -0.1 (-0.3 to 0.1) | -0.1 (-0.2 to -0.0) | -0.1 (-0.3 to 0.0) | 1.4 (0.7 to 2.7) |
| Complete-case analysis, carbamazepine vs. lamotrigine | 1,732 / 1,927 | -0.1 (-0.2 to -0.0) | -0.0 (-0.1 to 0.1) | -0.1 (-0.2 to -0.0) | -0.2 (-0.3 to -0.1) | 1.2 (0.8 to 1.9) |
| Singletons with no MCMs, carbamazepine vs. lamotrigine | 1,793 / 1,912 | -0.1 (-0.2 to -0.0) | -0.0 (-0.2 to 0.1) | -0.1 (-0.2 to -0.1) | -0.2 (-0.3 to -0.1) | 1.5 (0.9 to 2.3) |
| First infant per woman, carbamazepine vs. lamotrigine | 1,444 / 1,619 | -0.1 (-0.2 to -0.0) | -0.1 (-0.2 to 0.1) | -0.1 (-0.2 to -0.0) | -0.2 (-0.3 to -0.1) | 1.6 (1.0 to 2.6) |
| Female infants, carbamazepine vs. lamotrigine | 936 / 1,130 | -0.1 (-0.2 to -0.0) | -0.1 (-0.2 to 0.1) | -0.1 (-0.2 to -0.0) | -0.2 (-0.3 to -0.1) | 1.5 (0.8 to 3.0) |
| Male infants, carbamazepine vs. lamotrigine | 1,052 / 1,017 | -0.1 (-0.2 to 0.1) | 0.0 (-0.2 to 0.2) | -0.0 (-0.1 to 0.1) | -0.1 (-0.3 to 0.0) | 1.2 (0.7 to 2.1) |
| High vs. low dose of carbamazepine | 267 / 275 | -0.1 (-0.3 to 0.1) | -0.1 (-0.4 to 0.2) | -0.1 (-0.3 to 0.1) | -0.1 (-0.4 to 0.1) | 2.0 (0.7 to 5.6) |
| **Birth length z-score** | | | | | | |
| Use any time in pregnancy, carbamazepine vs. lamotrigine | 1,963 / 2,119 | -0.1 (-0.2 to -0.0) | -0.1 (-0.2 to 0.0) | -0.1 (-0.2 to 0.0) | -0.2 (-0.3 to -0.0) |  |
| Use in first trimester, carbamazepine vs. lamotrigine | 1,681 / 1,930 | -0.1 (-0.2 to -0.0) | -0.1 (-0.2 to 0.0) | -0.1 (-0.2 to -0.0) | -0.2 (-0.3 to -0.1) |  |
| Continuers, carbamazepine vs. lamotrigine | 461 / 1,006 | -0.2 (-0.3 to -0.1) | -0.2 (-0.4 to 0.0) | -0.2 (-0.3 to -0.1) | -0.2 (-0.4 to -0.1) |  |
| Mother with epilepsy, carbamazepine vs. lamotrigine | 1,655 / 1,441 | -0.1 (-0.2 to -0.0) | -0.1 (-0.3 to -0.0) | -0.1 (-0.2 to 0.0) | -0.1 (-0.3 to -0.0) |  |
| Mother with chronic pain, carbamazepine vs. lamotrigine | 260 / 542 | -0.2 (-0.4 to -0.0) | -0.1 (-0.3 to 0.2) | -0.2 (-0.4 to 0.1) | -0.3 (-0.6 to -0.0) |  |
| Monotherapy, carbamazepine vs. lamotrigine | 1,800 / 1,788 | -0.1 (-0.2 to 0.0) | -0.1 (-0.2 to 0.0) | -0.1 (-0.2 to 0.0) | -0.1 (-0.2 to 0.0) |  |
| Polytherapy, carbamazepine vs. lamotrigine | 163 / 331 | -0.3 (-0.5 to -0.1) | -0.2 (-0.5 to 0.1) | -0.2 (-0.5 to 0.0) | -0.6 (-0.8 to -0.3) |  |
| Definite exposure, carbamazepine vs. lamotrigine | 539 / 1,074 | -0.1 (-0.2 to -0.0) | -0.1 (-0.2 to 0.1) | -0.1 (-0.2 to 0.1) | -0.3 (-0.5 to -0.1) |  |
| Complete-case analysis, carbamazepine vs. lamotrigine | 1,711 / 1,900 | -0.1 (-0.2 to -0.0) | -0.1 (-0.2 to 0.0) | -0.1 (-0.2 to -0.0) | -0.2 (-0.3 to -0.1) |  |
| Singletons with no MCMs, carbamazepine vs. lamotrigine | 1,773 / 1,893 | -0.1 (-0.2 to -0.0) | -0.1 (-0.2 to 0.1) | -0.1 (-0.1 to 0.0) | -0.2 (-0.3 to -0.1) |  |
| First infant per woman, carbamazepine vs. lamotrigine | 1,424 / 1,598 | -0.1 (-0.2 to -0.0) | -0.1 (-0.2 to 0.0) | -0.1 (-0.2 to 0.0) | -0.1 (-0.3 to -0.0) |  |
| Female infants, carbamazepine vs. lamotrigine | 928 / 1,116 | -0.1 (-0.2 to -0.0) | -0.1 (-0.3 to 0.1) | -0.1 (-0.2 to 0.0) | -0.2 (-0.4 to -0.1) |  |
| Male infants, carbamazepine vs. lamotrigine | 1,035 / 1,003 | -0.1 (-0.2 to 0.0) | -0.1 (-0.2 to 0.1) | -0.1 (-0.2 to 0.0) | -0.1 (-0.3 to 0.1) |  |
| High vs. low dose of carbamazepine | 260 / 273 | -0.1 (-0.3 to 0.0) | -0.1 (-0.4 to 0.1) | -0.2 (-0.4 to -0.0) | -0.0 (-0.3 to 0.2) |  |
| **Birth head circumference z-score                                          Microcephaly** | | | | | | |
| Use any time in pregnancy, carbamazepine vs. lamotrigine | 1,883 / 2,096 | -0.2 (-0.3 to -0.1) | -0.2 (-0.3 to -0.0) | -0.2 (-0.3 to -0.1) | -0.2 (-0.3 to -0.1) | 1.2 (0.7 to 1.9) |
| Use in first trimester, carbamazepine vs. lamotrigine | 1,605 / 1,906 | -0.2 (-0.3 to -0.2) | -0.2 (-0.4 to -0.1) | -0.3 (-0.4 to -0.2) | -0.3 (-0.4 to -0.2) | 1.3 (0.8 to 2.1) |
| Continuers, carbamazepine vs. lamotrigine | 456 / 1,002 | -0.3 (-0.4 to -0.2) | -0.3 (-0.5 to -0.1) | -0.4 (-0.5 to -0.2) | -0.4 (-0.6 to -0.2) | 1.3 (0.6 to 3.3) |
| Mother with epilepsy, carbamazepine vs. lamotrigine | 1,585 / 1,421 | -0.2 (-0.3 to -0.1) | -0.2 (-0.4 to -0.1) | -0.2 (-0.3 to -0.1) | -0.2 (-0.3 to -0.1) | 1.2 (0.7 to 1.9) |
| Mother with chronic pain, carbamazepine vs. lamotrigine | 256 / 543 | -0.2 (-0.4 to -0.0) | -0.2 (-0.6 to 0.1) | -0.2 (-0.4 to -0.1) | 0.0 (-0.2 to 0.3) | 2.7 (0.8 to 9.1) |
| Monotherapy, carbamazepine vs. lamotrigine | 1,728 / 1,767 | -0.2 (-0.2 to -0.1) | -0.1 (-0.2 to 0.0) | -0.1 (-0.2 to -0.1) | -0.2 (-0.3 to -0.0) | 1.2 (0.7 to 2.0) |
| Polytherapy, carbamazepine vs. lamotrigine | 155 / 329 | -0.6 (-0.8 to -0.4) | -0.5 (-0.8 to -0.2) | -0.6 (-0.8 to -0.3) | -0.7 (-1.0 to -0.4) | 2.6 (0.9 to 7.3) |
| Definite exposure, carbamazepine vs. lamotrigine | 530 / 1,065 | -0.3 (-0.4 to -0.2) | -0.3 (-0.4 to -0.1) | -0.3 (-0.4 to -0.2) | -0.3 (-0.5 to -0.1) | 1.2 (0.6 to 2.4) |
| Complete-case analysis, carbamazepine vs. lamotrigine | 1,640 / 1,884 | -0.2 (-0.3 to -0.1) | -0.2 (-0.3 to -0.1) | -0.2 (-0.3 to -0.1) | -0.3 (-0.4 to -0.1) | 1.3 (0.8 to 2.1) |
| Singletons with no MCMs, carbamazepine vs. lamotrigine | 1,701 / 1,872 | -0.2 (-0.3 to -0.1) | -0.2 (-0.3 to -0.0) | -0.2 (-0.3 to -0.2) | -0.2 (-0.3 to -0.1) | 1.1 (0.7 to 1.8) |
| First infant per woman, carbamazepine vs. lamotrigine | 1,362 / 1,579 | -0.2 (-0.3 to -0.1) | -0.2 (-0.4 to -0.0) | -0.3 (-0.4 to -0.1) | -0.2 (-0.4 to -0.1) | 1.1 (0.7 to 1.8) |
| Female infants, carbamazepine vs. lamotrigine | 896 / 1,103 | -0.2 (-0.3 to -0.1) | -0.2 (-0.3 to 0.0) | -0.2 (-0.3 to -0.1) | -0.2 (-0.4 to -0.1) | 1.2 (0.6 to 2.3) |
| Male infants, carbamazepine vs. lamotrigine | 987 / 993 | -0.2 (-0.3 to -0.0) | -0.2 (-0.3 to 0.0) | -0.2 (-0.3 to -0.1) | -0.1 (-0.3 to 0.1) | 1.1 (0.6 to 2.1) |
| High vs. low dose of carbamazepine | 256 / 271 | -0.2 (-0.4 to 0.0) | -0.2 (-0.6 to 0.1) | -0.3 (-0.6 to -0.0) | -0.2 (-0.5 to 0.1) | Not applicable |
| **Birth weight (grams)** | | | | | | |
| Use any time in pregnancy, carbamazepine vs. lamotrigine | 1,988 / 2,147 | -69 (-112 to -26) | -83 (-165 to -1) | -50 (-91 to -9) | -78 (-139 to -17) |  |
| Use in first trimester, carbamazepine vs. lamotrigine | 1,699 / 1,953 | -87 (-133 to -40) | -98 (-182 to -15) | -56 (-98 to -15) | -83 (-150 to -15) |  |
| Continuers, carbamazepine vs. lamotrigine | 466 / 1,021 | -92 (-161 to -23) | -169 (-321 to -18) | -58 (-120 to 4) | -67 (-147 to 13) |  |
| Mother with epilepsy, carbamazepine vs. lamotrigine | 1,676 / 1,459 | -67 (-113 to -21) | -96 (-190 to -2) | -60 (-98 to -21) | -42 (-105 to 21) |  |
| Mother with chronic pain, carbamazepine vs. lamotrigine | 263 / 552 | -105 (-213 to 2) | -240 (-450 to -29) | -89 (-203 to 26) | -92 (-238 to 54) |  |
| Monotherapy, carbamazepine vs. lamotrigine | 1,821 / 1,808 | -55 (-102 to -9) | -49 (-140 to 42) | -37 (-81 to 8) | -73 (-146 to 1) |  |
| Polytherapy, carbamazepine vs. lamotrigine | 167 / 339 | -250 (-376 to -124) | -461 (-752 to -169) | -245 (-382 to -108) | -170 (-365 to 25) |  |
| Definite exposure, carbamazepine vs. lamotrigine | 542 / 1,083 | -95 (-161 to -29) | -134 (-264 to -4) | -53 (-117 to 12) | -77 (-166 to 12) |  |
| Complete-case analysis, carbamazepine vs. lamotrigine | 1,732 / 1,927 | -60 (-105 to -14) | -45 (-125 to 34) | -48 (-91 to -4) | -107 (-167 to -46) |  |
| Singletons with no MCMs, carbamazepine vs. lamotrigine | 1,793 / 1,912 | -76 (-119 to -33) | -66 (-144 to 13) | -46 (-91 to -2) | -81 (-146 to -17) |  |
| First infant per woman, carbamazepine vs. lamotrigine | 1,444 / 1,619 | -81 (-132 to -31) | -86 (-170 to -1) | -44 (-91 to 2) | -71 (-141 to -2) |  |
| Female infants, carbamazepine vs. lamotrigine | 936 / 1,130 | -87 (-147 to -27) | -130 (-253 to -7) | -61 (-118 to -5) | -95 (-181 to -9) |  |
| Male infants, carbamazepine vs. lamotrigine | 1,052 / 1,017 | -63 (-126 to -1) | -61 (-179 to 58) | -44 (-110 to 22) | -85 (-187 to 17) |  |
| High vs. low dose of carbamazepine | 267 / 275 | -170 (-288 to -52) | -347 (-606 to -88) | -85 (-203 to 34) | -183 (-340 to -26) |  |
| **Birth length (cm)** | | | | | | |
| Use any time in pregnancy, carbamazepine vs. lamotrigine | 1,963 / 2,119 | -0.3 (-0.5 to -0.1) | -0.6 (-1.0 to -0.2) | -0.0 (-0.1 to 0.1) | -0.1 (-0.4 to 0.1) |  |
| Use in first trimester, carbamazepine vs. lamotrigine | 1,681 / 1,930 | -0.4 (-0.6 to -0.2) | -0.9 (-1.3 to -0.5) | -0.0 (-0.2 to 0.2) | -0.0 (-0.2 to 0.2) |  |
| Continuers, carbamazepine vs. lamotrigine | 461 / 1,006 | -0.5 (-0.8 to -0.2) | -1.0 (-1.7 to -0.2) | -0.3 (-0.6 to -0.0) | -0.5 (-0.9 to -0.1) |  |
| Mother with epilepsy, carbamazepine vs. lamotrigine | 1,655 / 1,441 | -0.3 (-0.5 to -0.1) | -0.7 (-1.1 to -0.2) | -0.1 (-0.3 to 0.1) | -0.1 (-0.3 to 0.1) |  |
| Mother with chronic pain, carbamazepine vs. lamotrigine | 260 / 542 | -0.6 (-1.1 to -0.1) | -1.2 (-2.1 to -0.3) | -0.2 (-0.7 to 0.2) | -0.1 (-0.7 to 0.6) |  |
| Monotherapy, carbamazepine vs. lamotrigine | 1,800 / 1,788 | -0.3 (-0.5 to -0.1) | -0.4 (-0.8 to 0.1) | -0.0 (-0.1 to 0.1) | -0.1 (-0.4 to 0.1) |  |
| Polytherapy, carbamazepine vs. lamotrigine | 163 / 331 | -0.8 (-1.4 to -0.3) | -1.3 (-2.5 to -0.2) | -0.6 (-1.1 to -0.1) | -0.6 (-1.2 to -0.0) |  |
| Definite exposure, carbamazepine vs. lamotrigine | 539 / 1,074 | -0.5 (-0.7 to -0.2) | -1.0 (-1.7 to -0.3) | -0.2 (-0.4 to 0.1) | -0.4 (-0.7 to -0.1) |  |
| Complete-case analysis, carbamazepine vs. lamotrigine | 1,711 / 1,900 | -0.3 (-0.5 to -0.1) | -0.6 (-1.1 to -0.2) | -0.0 (-0.2 to 0.2) | -0.0 (-0.3 to 0.2) |  |
| Singletons with no MCMs, carbamazepine vs. lamotrigine | 1,773 / 1,893 | -0.3 (-0.5 to -0.1) | -0.4 (-0.8 to -0.1) | -0.0 (-0.1 to 0.1) | -0.2 (-0.4 to 0.1) |  |
| First infant per woman, carbamazepine vs. lamotrigine | 1,424 / 1,598 | -0.3 (-0.6 to -0.1) | -0.8 (-1.2 to -0.3) | -0.0 (-0.2 to 0.2) | -0.1 (-0.4 to 0.1) |  |
| Female infants, carbamazepine vs. lamotrigine | 928 / 1,116 | -0.4 (-0.6 to -0.1) | -1.0 (-1.5 to -0.4) | -0.0 (-0.2 to 0.2) | -0.2 (-0.6 to 0.1) |  |
| Male infants, carbamazepine vs. lamotrigine | 1,035 / 1,003 | -0.3 (-0.6 to -0.0) | -0.5 (-1.0 to 0.0) | -0.1 (-0.4 to 0.2) | -0.4 (-0.7 to -0.1) |  |
| High vs. low dose of carbamazepine | 260 / 273 | -0.7 (-1.2 to -0.1) | -1.3 (-2.6 to -0.0) | -0.7 (-1.2 to -0.1) | -0.1 (-0.7 to 0.5) |  |
| **Birth head circumference (cm)** | | | | | | |
| Use any time in pregnancy, carbamazepine vs. lamotrigine | 1,883 / 2,096 | -0.3 (-0.5 to -0.2) | -0.0 (-0.2 to 0.2) | -0.0 (-0.0 to 0.0) | -0.0 (-0.2 to 0.2) |  |
| Use in first trimester, carbamazepine vs. lamotrigine | 1,605 / 1,906 | -0.4 (-0.6 to -0.3) | -0.0 (-0.3 to 0.3) | -0.0 (-0.0 to 0.0) | -0.2 (-0.4 to 0.0) |  |
| Continuers, carbamazepine vs. lamotrigine | 456 / 1,002 | -0.5 (-0.7 to -0.3) | -0.3 (-0.7 to 0.1) | -0.0 (-0.2 to 0.2) | -0.4 (-0.7 to -0.1) |  |
| Mother with epilepsy, carbamazepine vs. lamotrigine | 1,585 / 1,421 | -0.3 (-0.5 to -0.2) | -0.0 (-0.3 to 0.3) | -0.0 (-0.0 to 0.0) | -0.2 (-0.4 to -0.0) |  |
| Mother with chronic pain, carbamazepine vs. lamotrigine | 256 / 543 | -0.3 (-0.6 to 0.0) | -0.8 (-1.5 to -0.0) | -0.1 (-0.4 to 0.2) | -0.0 (-0.4 to 0.3) |  |
| Monotherapy, carbamazepine vs. lamotrigine | 1,728 / 1,767 | -0.3 (-0.4 to -0.1) | -0.0 (-0.3 to 0.2) | -0.0 (-0.0 to 0.0) | -0.2 (-0.4 to 0.0) |  |
| Polytherapy, carbamazepine vs. lamotrigine | 155 / 329 | -1.0 (-1.3 to -0.6) | -1.4 (-2.3 to -0.5) | -1.0 (-1.4 to -0.6) | -1.1 (-1.6 to -0.6) |  |
| Definite exposure, carbamazepine vs. lamotrigine | 530 / 1,065 | -0.5 (-0.7 to -0.3) | -0.0 (-0.4 to 0.4) | -0.0 (-0.1 to 0.1) | -0.4 (-0.7 to -0.0) |  |
| Complete-case analysis, carbamazepine vs. lamotrigine | 1,640 / 1,884 | -0.3 (-0.5 to -0.2) | -0.0 (-0.3 to 0.3) | -0.0 (-0.0 to 0.0) | -0.3 (-0.4 to -0.1) |  |
| Singletons with no MCMs, carbamazepine vs. lamotrigine | 1,701 / 1,872 | -0.4 (-0.5 to -0.2) | -0.0 (-0.2 to 0.2) | -0.0 (-0.0 to 0.0) | -0.3 (-0.5 to -0.1) |  |
| First infant per woman, carbamazepine vs. lamotrigine | 1,362 / 1,579 | -0.4 (-0.5 to -0.2) | -0.0 (-0.3 to 0.3) | -0.0 (-0.0 to 0.0) | -0.4 (-0.6 to -0.2) |  |
| Female infants, carbamazepine vs. lamotrigine | 896 / 1,103 | -0.4 (-0.6 to -0.2) | -0.5 (-0.9 to -0.2) | -0.0 (-0.2 to 0.2) | -0.4 (-0.6 to -0.2) |  |
| Male infants, carbamazepine vs. lamotrigine | 987 / 993 | -0.3 (-0.5 to -0.1) | -0.3 (-0.6 to 0.0) | -0.0 (-0.2 to 0.1) | -0.2 (-0.5 to 0.1) |  |
| High vs. low dose of carbamazepine | 256 / 271 | -0.4 (-0.8 to -0.1) | -0.6 (-1.3 to 0.2) | -0.1 (-0.4 to 0.2) | -0.2 (-0.7 to 0.2) |  |

AED = antiepileptic drug; CI = confidence interval; MCM = major congenital malformation; SGA = small for gestational age

AED use was ascertained at any time in pregnancy, except where noted (indented rows). Analyses on continuers used data from deliveries in 2006-2013.

In analyses of carbamazepine vs. lamotrigine, the reference was lamotrigine in the same exposure window. In dose-response analyses, the reference was the bottom tertile of mean daily dose of carbamazepine (2006-2013). To facilitate convergence of quantile regression models for birth length and head circumference in cm, we implemented 1% jittering of birth length and head circumference by introducing random noise with uniform distribution and width 0.01 around the observation´s value. All results were adjusted for birth year, maternal age at delivery, education, country of origin, marital status, body mass index, smoking in current pregnancy, alcohol dependence, diabetes, hypertension, epilepsy, depression, bipolar disorder, migraine, chronic pain, and other psychiatric disorders. When the smallest cell count was < 5, we did not produce adjusted results ("not applicable"). Models restricted to polytherapy compared infants exposed to carbamazepine and another AED (except lamotrigine) with those exposed to lamotrigine and another AED (except carbamazepine).

1. Effect-measure modification analysis for the association between in-utero carbamazepine exposure and the endpoints duration of pregnancy and size at birth

|  | | | **Adjusted difference (95% CI)** | | |  |
| --- | --- | --- | --- | --- | --- | --- |
| **Potential EMM** | **Endpoint** | **Exposed to carbamazepine / lamotrigine** | **Carbamazepine** | **Potential EMM** | **Interaction term** | **P-value for interaction term** |
| Smoking in pregnancy | Pregnancy duration (days) | 1,975 / 2,123 | -1.0 (-2.1 to 0.1) | -0.8 (-2.5 to 1.0) | -2.2 (-4.8 to 0.4) | 0.091 |
|  | Birth weight z-score | 1,988 / 2,147 | -0.1 (-0.2 to -0.0) | -0.4 (-0.5 to -0.3) | -0.1 (-0.3 to 0.1) | 0.200 |
|  | Birth length z-score | 1,963 / 2,119 | -0.1 (-0.2 to -0.0) | -0.4 (-0.6 to -0.3) | -0.0 (-0.2 to 0.2) | 0.995 |
|  | Head circumference z-score | 1,883 / 2,096 | -0.2 (-0.3 to -0.1) | -0.3 (-0.5 to -0.2) | 0.0 (-0.1 to 0.2) | 0.663 |
|  | Birth weight (grams) | 1,988 / 2,147 | -55 (-100 to -10) | -162 (-235 to -89) | -110 (-218 to -3) | 0.045 |
|  | Birth length (cm) | 1,963 / 2,119 | -0.2 (-0.4 to -0.0) | -0.8 (-1.1 to -0.4) | -0.4 (-0.8 to 0.1) | 0.146 |
|  | Head circumference (cm) | 1,883 / 2,096 | -0.3 (-0.4 to -0.2) | -0.4 (-0.6 to -0.1) | -0.2 (-0.5 to 0.1) | 0.276 |
| Use of SSRIs in pregnancy | Pregnancy duration (days) | 1,975 / 2,123 | -1.0 (-2.1 to 0.0) | -1.6 (-3.5 to 0.4) | -5.8 (-9.7 to -2.0) | 0.003 |
|  | Birth weight z-score | 1,988 / 2,147 | -0.1 (-0.2 to -0.0) | -0.0 (-0.2 to 0.1) | -0.0 (-0.3 to 0.3) | 0.943 |
|  | Birth length z-score | 1,963 / 2,119 | -0.1 (-0.2 to -0.0) | -0.3 (-0.4 to -0.1) | 0.1 (-0.2 to 0.3) | 0.596 |
|  | Head circumference z-score | 1,883 / 2,096 | -0.2 (-0.3 to -0.1) | -0.1 (-0.2 to 0.1) | 0.0 (-0.3 to 0.3) | 0.991 |
|  | Birth weight (grams) | 1,988 / 2,147 | -59 (-103 to -15) | -35 (-115 to 46) | -197 (-357 to -38) | 0.015 |
|  | Birth length (cm) | 1,963 / 2,119 | -0.3 (-0.5 to -0.1) | -0.5 (-0.9 to -0.2) | -0.9 (-1.6 to -0.2) | 0.016 |
|  | Head circumference (cm) | 1,883 / 2,096 | -0.3 (-0.4 to -0.2) | -0.1 (-0.3 to 0.2) | -0.6 (-1.1 to -0.2) | 0.009 |

AED = antiepileptic drug; CI = confidence interval; EMM = effect-measure modifier; SSRI = selective serotonin reuptake inhibitor.

AED use was ascertained at any time in pregnancy. The reference was lamotrigine in the same exposure window. Results were obtained with linear regression analysis models and were adjusted for birth year, maternal age at delivery, education, country of origin, marital status, body mass index, smoking in current pregnancy, alcohol dependence, diabetes, hypertension, epilepsy, depression, bipolar disorder, migraine, chronic pain, and other psychiatric disorders.

1. Association between in-utero pregabalin exposure and the endpoints duration of pregnancy and size at birth

|  | | **Difference (95% CI)** | | | | **Odds ratio** |
| --- | --- | --- | --- | --- | --- | --- |
|  | | | **At percentile** | | | **(95% CI)** |
|  | **Exposed to pregabalin/ reference** | **Mean** | **10^th^** | **50^th^** | **90^th^** |  |
| **Pregnancy duration (days)                                                Preterm birth** | | | | | | |
| Use any time in pregnancy, pregabalin vs. lamotrigine | 522 / 2,190 | -1.1 (-3.0 to 0.8) | -2.7 (-6.7 to 1.2) | -0.5 (-2.5 to 1.4) | 0.3 (-1.6 to 2.3) | 1.5 (1.0 to 2.4) |
| Use in first trimester, pregabalin vs. lamotrigine | 484 / 1,977 | -1.8 (-3.7 to 0.2) | -3.2 (-7.5 to 1.1) | -0.5 (-2.4 to 1.3) | -0.1 (-2.2 to 2.0) | 1.9 (1.2 to 3.0) |
| Continuers, pregabalin vs. lamotrigine | 142 / 1,025 | -1.2 (-4.7 to 2.3) | -0.5 (-7.2 to 6.2) | 0.4 (-3.4 to 4.2) | -0.6 (-4.7 to 3.5) | 2.3 (1.0 to 5.3) |
| Mother with epilepsy, pregabalin vs. lamotrigine | 33 / 1,537 | -5.6 (-10.7 to -0.4) | -11.2 (-35.7 to 13.3) | -4.2 (-10.0 to 1.6) | 3.3 (-4.7 to 11.2) | 4.2 (1.6 to 11.4) |
| Mother with chronic pain, pregabalin vs. lamotrigine | 364 / 544 | -1.0 (-3.8 to 1.9) | -3.1 (-8.1 to 1.9) | -0.7 (-2.9 to 1.5) | 0.0 (-3.2 to 3.2) | 1.9 (1.1 to 3.4) |
| Monotherapy, pregabalin vs. lamotrigine | 492 / 1,787 | -0.7 (-2.7 to 1.3) | -1.5 (-5.5 to 2.5) | -0.5 (-2.4 to 1.4) | -0.1 (-2.3 to 2.0) | 1.5 (0.9 to 2.5) |
| Polytherapy, pregabalin vs. lamotrigine | 30 / 403 | -1.1 (-8.3 to 6.1) | 2.8 (-17.5 to 23.2) | 1.5 (-6.1 to 9.1) | -5.6 (-14.8 to 3.7) | 0.7 (0.2 to 3.2) |
| Definite exposure, pregabalin vs. lamotrigine | 163 / 1,086 | -0.2 (-3.3 to 3.0) | -1.4 (-9.4 to 6.6) | 1.0 (-1.8 to 3.8) | 0.5 (-3.7 to 4.7) | 1.6 (0.7 to 3.8) |
| Complete-case analysis, pregabalin vs. lamotrigine | 469 / 1,965 | -1.0 (-3.0 to 1.1) | -2.5 (-6.6 to 1.6) | -0.8 (-3.1 to 1.5) | -0.0 (-2.4 to 2.4) | 1.5 (0.9 to 2.4) |
| Singletons with no MCMs, pregabalin vs. lamotrigine | 477 / 1,974 | -0.8 (-2.6 to 1.1) | -1.2 (-4.6 to 2.3) | -0.7 (-2.5 to 1.1) | 0.5 (-2.0 to 3.0) | 1.6 (1.0 to 2.6) |
| First infant per woman, pregabalin vs. lamotrigine | 507 / 1,656 | -1.1 (-3.1 to 0.9) | -2.0 (-6.1 to 2.1) | -1.0 (-2.9 to 0.9) | -0.4 (-2.6 to 1.8) | 1.4 (0.9 to 2.3) |
| Female infants, pregabalin vs. lamotrigine | 265 / 1,146 | -2.0 (-4.6 to 0.7) | -3.2 (-8.7 to 2.2) | -1.9 (-4.2 to 0.5) | -2.4 (-5.1 to 0.3) | 1.9 (1.0 to 3.4) |
| Male infants, pregabalin vs. lamotrigine | 257 / 1,044 | -0.2 (-3.1 to 2.6) | 0.4 (-4.6 to 5.4) | -0.2 (-3.5 to 3.1) | 1.2 (-1.7 to 4.2) | 1.2 (0.6 to 2.4) |
| High vs. low dose of pregabalin | 175 / 174 | 0.6 (-2.7 to 3.9) | 0.4 (-7.0 to 7.7) | 1.1 (-1.9 to 4.2) | 1.2 (-2.3 to 4.7) | 1.1 (0.6 to 2.3) |
| **Birth weight z-score                                                                SGA** | | | | | | |
| Use any time in pregnancy, pregabalin vs. lamotrigine | 528 / 2,215 | -0.1 (-0.3 to 0.0) | -0.0 (-0.3 to 0.2) | -0.2 (-0.3 to 0.0) | -0.2 (-0.4 to 0.0) | 1.3 (0.6 to 3.0) |
| Use in first trimester, pregabalin vs. lamotrigine | 489 / 2,001 | -0.2 (-0.3 to -0.0) | -0.0 (-0.3 to 0.2) | -0.2 (-0.4 to -0.0) | -0.2 (-0.5 to -0.0) | 1.3 (0.6 to 3.1) |
| Continuers, pregabalin vs. lamotrigine | 142 / 1,033 | -0.1 (-0.4 to 0.1) | -0.2 (-0.7 to 0.3) | -0.1 (-0.4 to 0.2) | -0.2 (-0.6 to 0.1) | 0.6 (0.1 to 3.0) |
| Mother with epilepsy, pregabalin vs. lamotrigine | 33 / 1,550 | 0.1 (-0.3 to 0.5) | 0.2 (-0.9 to 1.2) | 0.2 (-0.2 to 0.6) | -0.3 (-0.7 to 0.2) | Not applicable |
| Mother with chronic pain, pregabalin vs. lamotrigine | 369 / 555 | -0.1 (-0.3 to 0.1) | -0.0 (-0.4 to 0.3) | -0.1 (-0.3 to 0.1) | -0.3 (-0.6 to -0.0) | 1.5 (0.5 to 4.1) |
| Monotherapy, pregabalin vs. lamotrigine | 498 / 1,808 | -0.1 (-0.3 to 0.0) | -0.0 (-0.3 to 0.3) | -0.1 (-0.3 to 0.0) | -0.2 (-0.4 to -0.0) | 1.7 (0.7 to 4.0) |
| Polytherapy, pregabalin vs. lamotrigine | 30 / 407 | -0.0 (-0.5 to 0.5) | 0.7 (0.0 to 1.4) | 0.2 (-0.4 to 0.8) | -0.0 (-0.9 to 0.8) | Not applicable |
| Definite exposure, pregabalin vs. lamotrigine | 163 / 1,097 | -0.1 (-0.3 to 0.2) | 0.0 (-0.4 to 0.4) | -0.1 (-0.4 to 0.2) | -0.2 (-0.6 to 0.1) | 1.0 (0.2 to 4.0) |
| Complete-case analysis, pregabalin vs. lamotrigine | 475 / 1,983 | -0.1 (-0.3 to 0.0) | -0.0 (-0.3 to 0.3) | -0.1 (-0.3 to 0.0) | -0.2 (-0.4 to 0.0) | 1.2 (0.5 to 2.8) |
| Singletons with no MCMs, pregabalin vs. lamotrigine | 476 / 1,970 | -0.1 (-0.3 to 0.0) | 0.0 (-0.2 to 0.2) | -0.2 (-0.3 to -0.0) | -0.2 (-0.4 to -0.0) | 1.5 (0.6 to 3.5) |
| First infant per woman, pregabalin vs. lamotrigine | 506 / 1,651 | -0.1 (-0.2 to 0.0) | -0.0 (-0.3 to 0.3) | -0.1 (-0.3 to 0.0) | -0.2 (-0.4 to -0.0) | 1.5 (0.7 to 3.6) |
| Female infants, pregabalin vs. lamotrigine | 270 / 1,159 | -0.1 (-0.3 to 0.1) | 0.1 (-0.3 to 0.5) | -0.1 (-0.4 to 0.1) | -0.3 (-0.5 to 0.0) | 1.9 (0.4 to 8.3) |
| Male infants, pregabalin vs. lamotrigine | 258 / 1,056 | -0.1 (-0.3 to 0.1) | 0.0 (-0.3 to 0.3) | -0.2 (-0.4 to 0.0) | -0.1 (-0.5 to 0.2) | 1.4 (0.5 to 4.2) |
| High vs. low dose of pregabalin | 177 / 176 | 0.0 (-0.2 to 0.3) | -0.3 (-0.8 to 0.1) | 0.2 (-0.1 to 0.5) | 0.2 (-0.1 to 0.6) | 1.3 (0.4 to 4.6) |
| **Birth length z-score** | | | | | | |
| Use any time in pregnancy, pregabalin vs. lamotrigine | 521 / 2,186 | -0.1 (-0.2 to 0.0) | -0.0 (-0.3 to 0.2) | -0.1 (-0.3 to 0.0) | -0.1 (-0.4 to 0.1) |  |
| Use in first trimester, pregabalin vs. lamotrigine | 484 / 1,977 | -0.1 (-0.2 to 0.0) | -0.1 (-0.4 to 0.1) | -0.2 (-0.4 to 0.0) | -0.1 (-0.3 to 0.2) |  |
| Continuers, pregabalin vs. lamotrigine | 140 / 1,018 | -0.1 (-0.4 to 0.1) | -0.1 (-0.5 to 0.4) | -0.1 (-0.4 to 0.3) | -0.2 (-0.7 to 0.2) |  |
| Mother with epilepsy, pregabalin vs. lamotrigine | 32 / 1,530 | -0.1 (-0.5 to 0.3) | -0.1 (-0.9 to 0.8) | 0.1 (-0.3 to 0.6) | -0.1 (-0.7 to 0.5) |  |
| Mother with chronic pain, pregabalin vs. lamotrigine | 364 / 546 | -0.2 (-0.3 to 0.0) | -0.0 (-0.3 to 0.3) | -0.1 (-0.3 to 0.1) | -0.3 (-0.6 to -0.0) |  |
| Monotherapy, pregabalin vs. lamotrigine | 491 / 1,788 | -0.1 (-0.2 to 0.0) | -0.1 (-0.3 to 0.0) | -0.1 (-0.3 to 0.1) | -0.2 (-0.4 to 0.0) |  |
| Polytherapy, pregabalin vs. lamotrigine | 30 / 398 | -0.0 (-0.5 to 0.4) | 0.3 (-0.6 to 1.2) | -0.1 (-0.7 to 0.5) | -0.3 (-1.1 to 0.5) |  |
| Definite exposure, pregabalin vs. lamotrigine | 160 / 1,087 | 0.1 (-0.2 to 0.3) | -0.0 (-0.4 to 0.3) | 0.0 (-0.3 to 0.4) | 0.1 (-0.4 to 0.6) |  |
| Complete-case analysis, pregabalin vs. lamotrigine | 468 / 1,955 | -0.1 (-0.2 to 0.1) | -0.0 (-0.2 to 0.2) | -0.1 (-0.3 to 0.1) | -0.1 (-0.4 to 0.1) |  |
| Singletons with no MCMs, pregabalin vs. lamotrigine | 471 / 1,950 | -0.1 (-0.2 to 0.0) | -0.0 (-0.3 to 0.2) | -0.1 (-0.3 to 0.1) | -0.1 (-0.4 to 0.2) |  |
| First infant per woman, pregabalin vs. lamotrigine | 500 / 1,629 | -0.1 (-0.2 to 0.1) | -0.0 (-0.2 to 0.2) | -0.0 (-0.2 to 0.2) | -0.2 (-0.5 to 0.1) |  |
| Female infants, pregabalin vs. lamotrigine | 266 / 1,144 | -0.1 (-0.2 to 0.1) | -0.1 (-0.4 to 0.2) | -0.0 (-0.2 to 0.2) | -0.0 (-0.3 to 0.3) |  |
| Male infants, pregabalin vs. lamotrigine | 255 / 1,042 | -0.1 (-0.3 to 0.1) | 0.1 (-0.2 to 0.3) | -0.2 (-0.5 to 0.1) | -0.3 (-0.7 to 0.1) |  |
| High vs. low dose of pregabalin | 172 / 175 | 0.0 (-0.2 to 0.2) | -0.3 (-0.7 to 0.0) | -0.0 (-0.3 to 0.2) | 0.1 (-0.2 to 0.5) |  |
| **Birth head circumference z-score                                               Microcephaly** | | | | | | |
| Use any time in pregnancy, pregabalin vs. lamotrigine | 516 / 2,160 | -0.0 (-0.1 to 0.1) | 0.1 (-0.1 to 0.3) | -0.0 (-0.2 to 0.1) | -0.1 (-0.3 to 0.1) | 1.2 (0.5 to 2.9) |
| Use in first trimester, pregabalin vs. lamotrigine | 480 / 1,951 | -0.0 (-0.2 to 0.1) | 0.1 (-0.1 to 0.4) | -0.1 (-0.2 to 0.1) | 0.0 (-0.2 to 0.3) | 1.3 (0.5 to 3.4) |
| Continuers, pregabalin vs. lamotrigine | 136 / 1,012 | -0.1 (-0.3 to 0.2) | -0.1 (-0.7 to 0.6) | -0.0 (-0.3 to 0.3) | -0.1 (-0.5 to 0.2) | 5.3 (0.9 to 30.8) |
| Mother with epilepsy, pregabalin vs. lamotrigine | 32 / 1,508 | -0.0 (-0.4 to 0.4) | 0.0 (-1.0 to 1.1) | 0.1 (-0.3 to 0.4) | 0.4 (-0.5 to 1.3) | Not applicable |
| Mother with chronic pain, pregabalin vs. lamotrigine | 363 / 545 | -0.0 (-0.2 to 0.1) | -0.0 (-0.4 to 0.4) | -0.1 (-0.3 to 0.1) | -0.2 (-0.5 to 0.1) | 0.5 (0.1 to 1.8) |
| Monotherapy, pregabalin vs. lamotrigine | 487 / 1,767 | -0.0 (-0.2 to 0.1) | 0.2 (-0.0 to 0.4) | -0.1 (-0.2 to 0.1) | -0.0 (-0.3 to 0.2) | 0.8 (0.3 to 2.1) |
| Polytherapy, pregabalin vs. lamotrigine | 29 / 393 | -0.0 (-0.6 to 0.5) | -0.3 (-1.2 to 0.7) | -0.0 (-0.5 to 0.5) | 0.6 (-0.4 to 1.6) | Not applicable |
| Definite exposure, pregabalin vs. lamotrigine | 160 / 1,077 | 0.1 (-0.2 to 0.4) | 0.4 (-0.1 to 0.8) | 0.2 (-0.2 to 0.6) | -0.1 (-0.5 to 0.3) | Not applicable |
| Complete-case analysis, pregabalin vs. lamotrigine | 465 / 1,936 | -0.0 (-0.2 to 0.1) | 0.1 (-0.1 to 0.4) | -0.0 (-0.2 to 0.1) | -0.1 (-0.3 to 0.1) | 1.0 (0.4 to 2.7) |
| Singletons with no MCMs, pregabalin vs. lamotrigine | 468 / 1,927 | -0.0 (-0.2 to 0.1) | 0.2 (-0.1 to 0.5) | -0.1 (-0.3 to 0.0) | -0.1 (-0.3 to 0.1) | 1.0 (0.4 to 2.5) |
| First infant per woman, pregabalin vs. lamotrigine | 496 / 1,610 | 0.0 (-0.1 to 0.2) | 0.1 (-0.2 to 0.3) | -0.0 (-0.2 to 0.1) | -0.0 (-0.2 to 0.1) | 1.2 (0.5 to 2.9) |
| Female infants, pregabalin vs. lamotrigine | 264 / 1,128 | 0.0 (-0.2 to 0.2) | 0.3 (-0.1 to 0.6) | -0.1 (-0.3 to 0.1) | 0.0 (-0.3 to 0.3) | 1.2 (0.3 to 4.5) |
| Male infants, pregabalin vs. lamotrigine | 252 / 1,032 | -0.0 (-0.3 to 0.2) | -0.3 (-0.6 to 0.1) | 0.0 (-0.2 to 0.2) | -0.1 (-0.4 to 0.2) | 1.6 (0.5 to 5.7) |
| High vs. low dose of pregabalin | 170 / 174 | 0.0 (-0.2 to 0.2) | 0.1 (-0.2 to 0.5) | 0.1 (-0.2 to 0.4) | -0.3 (-0.8 to 0.1) | Not applicable |
| **Birth weight (grams)** | | | | | | |
| Use any time in pregnancy, pregabalin vs. lamotrigine | 528 / 2,215 | -83 (-163 to -3) | -94 (-252 to 63) | -104 (-181 to -26) | -108 (-242 to 27) |  |
| Use in first trimester, pregabalin vs. lamotrigine | 489 / 2,001 | -127 (-210 to -44) | -114 (-283 to 56) | -113 (-200 to -27) | -113 (-244 to 18) |  |
| Continuers, pregabalin vs. lamotrigine | 142 / 1,033 | -128 (-276 to 19) | -40 (-351 to 272) | -169 (-363 to 24) | -107 (-336 to 121) |  |
| Mother with epilepsy, pregabalin vs. lamotrigine | 33 / 1,550 | -183 (-402 to 35) | -109 (-901 to 683) | -72 (-326 to 183) | -347 (-709 to 14) |  |
| Mother with chronic pain, pregabalin vs. lamotrigine | 369 / 555 | -98 (-212 to 16) | -134 (-338 to 70) | -136 (-239 to -33) | -157 (-312 to -3) |  |
| Monotherapy, pregabalin vs. lamotrigine | 498 / 1,808 | -75 (-159 to 10) | -34 (-215 to 147) | -97 (-184 to -10) | -82 (-211 to 47) |  |
| Polytherapy, pregabalin vs. lamotrigine | 30 / 407 | -76 (-349 to 197) | 226 (-336 to 788) | -30 (-332 to 272) | -235 (-738 to 268) |  |
| Definite exposure, pregabalin vs. lamotrigine | 163 / 1,097 | -47 (-183 to 90) | 151 (-80 to 382) | -91 (-226 to 45) | -126 (-348 to 97) |  |
| Complete-case analysis, pregabalin vs. lamotrigine | 475 / 1,983 | -80 (-165 to 5) | -52 (-221 to 117) | -129 (-223 to -36) | -68 (-203 to 67) |  |
| Singletons with no MCMs, pregabalin vs. lamotrigine | 476 / 1,970 | -92 (-170 to -15) | -102 (-234 to 31) | -89 (-169 to -10) | -89 (-232 to 54) |  |
| First infant per woman, pregabalin vs. lamotrigine | 506 / 1,651 | -85 (-164 to -5) | -75 (-216 to 66) | -95 (-186 to -4) | -95 (-205 to 15) |  |
| Female infants, pregabalin vs. lamotrigine | 270 / 1,159 | -111 (-220 to -2) | -142 (-392 to 108) | -126 (-223 to -30) | -169 (-333 to -4) |  |
| Male infants, pregabalin vs. lamotrigine | 258 / 1,056 | -59 (-176 to 58) | -41 (-231 to 148) | -22 (-138 to 94) | -173 (-324 to -22) |  |
| High vs. low dose of pregabalin | 177 / 176 | 24 (-106 to 154) | 23 (-273 to 319) | 25 (-100 to 150) | -37 (-256 to 181) |  |
| **Birth length (cm)** | | | | | | |
| Use any time in pregnancy, pregabalin vs. lamotrigine | 521 / 2,186 | -0.4 (-0.7 to 0.0) | -0.0 (-0.6 to 0.6) | -0.0 (-0.3 to 0.3) | -0.7 (-1.2 to -0.1) |  |
| Use in first trimester, pregabalin vs. lamotrigine | 484 / 1,977 | -0.4 (-0.8 to -0.1) | -0.0 (-0.7 to 0.6) | -0.0 (-0.3 to 0.3) | -0.8 (-1.3 to -0.2) |  |
| Continuers, pregabalin vs. lamotrigine | 140 / 1,018 | -0.6 (-1.3 to 0.0) | -1.0 (-2.2 to 0.2) | -0.2 (-1.0 to 0.5) | -0.9 (-2.1 to 0.4) |  |
| Mother with epilepsy, pregabalin vs. lamotrigine | 32 / 1,530 | -0.9 (-1.9 to 0.0) | -1.5 (-4.8 to 1.8) | -0.0 (-0.9 to 0.9) | -1.5 (-3.6 to 0.6) |  |
| Mother with chronic pain, pregabalin vs. lamotrigine | 364 / 546 | -0.5 (-1.0 to 0.0) | -0.5 (-1.4 to 0.3) | -0.4 (-0.9 to 0.0) | -0.6 (-1.4 to 0.1) |  |
| Monotherapy, pregabalin vs. lamotrigine | 491 / 1,788 | -0.3 (-0.7 to 0.1) | -0.0 (-0.7 to 0.7) | -0.0 (-0.3 to 0.3) | -0.7 (-1.3 to -0.2) |  |
| Polytherapy, pregabalin vs. lamotrigine | 30 / 398 | -0.4 (-1.6 to 0.8) | -0.0 (-2.9 to 2.9) | -0.6 (-1.7 to 0.5) | -1.4 (-3.3 to 0.5) |  |
| Definite exposure, pregabalin vs. lamotrigine | 160 / 1,087 | 0.0 (-0.6 to 0.6) | 0.1 (-0.8 to 0.9) | 0.0 (-0.5 to 0.5) | 0.4 (-0.6 to 1.4) |  |
| Complete-case analysis, pregabalin vs. lamotrigine | 468 / 1,955 | -0.3 (-0.7 to 0.0) | -0.0 (-0.6 to 0.6) | -0.3 (-0.6 to 0.0) | -0.4 (-1.0 to 0.2) |  |
| Singletons with no MCMs, pregabalin vs. lamotrigine | 471 / 1,950 | -0.3 (-0.6 to 0.1) | -0.0 (-0.5 to 0.5) | -0.2 (-0.5 to 0.1) | -0.7 (-1.3 to -0.1) |  |
| First infant per woman, pregabalin vs. lamotrigine | 500 / 1,629 | -0.3 (-0.7 to 0.0) | -0.0 (-0.6 to 0.6) | -0.2 (-0.5 to 0.1) | -0.6 (-1.2 to -0.0) |  |
| Female infants, pregabalin vs. lamotrigine | 266 / 1,144 | -0.3 (-0.8 to 0.1) | -0.9 (-2.0 to 0.1) | -0.3 (-0.7 to 0.1) | -0.7 (-1.3 to -0.0) |  |
| Male infants, pregabalin vs. lamotrigine | 255 / 1,042 | -0.4 (-0.9 to 0.2) | -0.0 (-0.8 to 0.8) | -0.3 (-0.8 to 0.2) | -0.6 (-1.5 to 0.2) |  |
| High vs. low dose of pregabalin | 172 / 175 | -0.0 (-0.6 to 0.6) | -0.3 (-1.4 to 0.9) | -0.0 (-0.5 to 0.5) | 0.5 (-0.3 to 1.2) |  |
| **Birth head circumference (cm)** | | | | | | |
| Use any time in pregnancy, pregabalin vs. lamotrigine | 516 / 2,160 | -0.0 (-0.3 to 0.2) | -0.0 (-0.3 to 0.2) | -0.0 (-0.1 to 0.0) | -0.0 (-0.3 to 0.3) |  |
| Use in first trimester, pregabalin vs. lamotrigine | 480 / 1,951 | -0.2 (-0.4 to 0.1) | -0.0 (-0.4 to 0.4) | -0.0 (-0.2 to 0.2) | -0.0 (-0.3 to 0.3) |  |
| Continuers, pregabalin vs. lamotrigine | 136 / 1,012 | -0.3 (-0.8 to 0.1) | -1.0 (-1.9 to -0.1) | -0.0 (-0.5 to 0.5) | -0.1 (-0.7 to 0.4) |  |
| Mother with epilepsy, pregabalin vs. lamotrigine | 32 / 1,508 | -0.5 (-1.1 to 0.2) | -2.0 (-3.8 to -0.2) | -0.0 (-0.1 to 0.1) | -1.0 (-1.8 to -0.1) |  |
| Mother with chronic pain, pregabalin vs. lamotrigine | 363 / 545 | -0.1 (-0.4 to 0.3) | 0.2 (-0.3 to 0.8) | -0.3 (-0.7 to 0.0) | -0.4 (-0.8 to 0.1) |  |
| Monotherapy, pregabalin vs. lamotrigine | 487 / 1,767 | -0.0 (-0.3 to 0.3) | 0.0 (-0.4 to 0.4) | -0.0 (-0.2 to 0.2) | -0.3 (-0.6 to 0.0) |  |
| Polytherapy, pregabalin vs. lamotrigine | 29 / 393 | -0.3 (-1.1 to 0.6) | -0.6 (-2.5 to 1.4) | -0.0 (-1.0 to 1.0) | -0.0 (-1.7 to 1.7) |  |
| Definite exposure, pregabalin vs. lamotrigine | 160 / 1,077 | 0.1 (-0.3 to 0.5) | 0.4 (-0.3 to 1.0) | -0.0 (-0.5 to 0.4) | -0.4 (-1.0 to 0.2) |  |
| Complete-case analysis, pregabalin vs. lamotrigine | 465 / 1,936 | -0.1 (-0.3 to 0.2) | -0.0 (-0.4 to 0.4) | -0.0 (-0.0 to 0.0) | -0.0 (-0.3 to 0.3) |  |
| Singletons with no MCMs, pregabalin vs. lamotrigine | 468 / 1,927 | -0.1 (-0.3 to 0.2) | 0.0 (-0.4 to 0.4) | -0.0 (-0.0 to 0.0) | -0.3 (-0.6 to 0.0) |  |
| First infant per woman, pregabalin vs. lamotrigine | 496 / 1,610 | -0.0 (-0.3 to 0.2) | -0.0 (-0.3 to 0.3) | -0.0 (-0.2 to 0.2) | -0.3 (-0.7 to -0.0) |  |
| Female infants, pregabalin vs. lamotrigine | 264 / 1,128 | -0.1 (-0.4 to 0.3) | -0.1 (-0.7 to 0.5) | -0.3 (-0.6 to 0.1) | -0.3 (-0.8 to 0.2) |  |
| Male infants, pregabalin vs. lamotrigine | 252 / 1,032 | -0.0 (-0.4 to 0.3) | 0.1 (-0.5 to 0.8) | -0.0 (-0.4 to 0.4) | -0.3 (-0.7 to 0.2) |  |
| High vs. low dose of pregabalin | 170 / 174 | -0.1 (-0.5 to 0.3) | 0.0 (-0.8 to 0.8) | 0.0 (-0.4 to 0.5) | -0.4 (-1.0 to 0.1) |  |

AED = antiepileptic drug; CI = confidence interval; MCM = major congenital malformation; SGA = small for gestational age

AED use was ascertained at any time in pregnancy, except where noted (indented rows). Analyses on continuers used data from deliveries in 2006-2013. In analyses of carbamazepine vs. lamotrigine, the reference was lamotrigine in the same exposure window. In dose-response analyses, the reference was the bottom tertile of mean daily dose of carbamazepine (2006-2013). To facilitate convergence of quantile regression models for birth length and head circumference in cm, we implemented 1% jittering of birth length and head circumference by introducing random noise with uniform distribution and width 0.01 around the observation´s value. All results were adjusted for birth year, maternal age at delivery, education, country of origin, marital status, body mass index, smoking in current pregnancy, alcohol dependence, diabetes, hypertension, epilepsy, depression, bipolar disorder, migraine, chronic pain, and other psychiatric disorders. When the smallest cell count was < 5, we did not produce adjusted results ("not applicable"). Models restricted to polytherapy compared infants exposed to carbamazepine and another AED (except lamotrigine) with those exposed to lamotrigine and another AED (except carbamazepine).

1. Effect-measure modification analysis for the association between in-utero pregabalin exposure and the endpoints duration of pregnancy and size at birth

|  | | | **Adjusted difference (95% CI)** | | |  |
| --- | --- | --- | --- | --- | --- | --- |
| **Potential EMM** | **Endpoint** | **Exposed to pregabalin / lamotrigine** | **Pregabalin** | **Potential EMM** | **Interaction term** | **P-value for interaction term** |
| Smoking in pregnancy | Pregnancy duration (days) | 522 / 2,190 | -1.4 (-3.7 to 0.8) | -1.5 (-3.2 to 0.2) | 0.9 (-2.1 to 3.9) | 0.544 |
|  | Birth weight z-score | 528 / 2,215 | -0.1 (-0.2 to 0.1) | -0.4 (-0.6 to -0.3) | -0.1 (-0.4 to 0.1) | 0.185 |
|  | Birth length z-score | 521 / 2,186 | -0.1 (-0.2 to 0.1) | -0.5 (-0.6 to -0.3) | -0.1 (-0.3 to 0.1) | 0.341 |
|  | Head circumference z-score | 516 / 2,160 | 0.0 (-0.1 to 0.2) | -0.4 (-0.5 to -0.2) | -0.1 (-0.3 to 0.2) | 0.517 |
|  | Birth weight (grams) | 528 / 2,215 | -68 (-161 to 24) | -189 (-260 to -117) | -40 (-164 to 84) | 0.528 |
|  | Birth length (cm) | 521 / 2,186 | -0.3 (-0.7 to 0.1) | -0.9 (-1.2 to -0.6) | -0.2 (-0.7 to 0.4) | 0.591 |
|  | Head circumference (cm) | 516 / 2,160 | -0.0 (-0.3 to 0.3) | -0.5 (-0.7 to -0.2) | -0.1 (-0.5 to 0.3) | 0.595 |
| Use of SSRIs in pregnancy | Pregnancy duration (days) | 522 / 2,190 | -1.9 (-4.1 to 0.3) | -2.4 (-4.4 to -0.5) | 2.0 (-1.2 to 5.2) | 0.211 |
|  | Birth weight z-score | 528 / 2,215 | -0.1 (-0.3 to 0.0) | -0.0 (-0.2 to 0.1) | 0.1 (-0.2 to 0.3) | 0.677 |
|  | Birth length z-score | 521 / 2,186 | -0.2 (-0.3 to -0.0) | -0.2 (-0.4 to -0.1) | 0.2 (-0.1 to 0.4) | 0.156 |
|  | Head circumference z-score | 516 / 2,160 | 0.0 (-0.2 to 0.2) | -0.1 (-0.2 to 0.1) | -0.1 (-0.3 to 0.2) | 0.673 |
|  | Birth weight (grams) | 528 / 2,215 | -105 (-197 to -14) | -57 (-137 to 23) | 59 (-73 to 192) | 0.382 |
|  | Birth length (cm) | 521 / 2,186 | -0.5 (-0.9 to -0.1) | -0.6 (-1.0 to -0.3) | 0.4 (-0.1 to 1.0) | 0.137 |
|  | Head circumference (cm) | 516 / 2,160 | -0.0 (-0.3 to 0.2) | -0.1 (-0.4 to 0.1) | -0.0 (-0.5 to 0.4) | 0.828 |

AED = antiepileptic drug; CI = confidence interval; EMM = effect-measure modifier; SSRI = selective serotonin reuptake inhibitor.

AED use was ascertained at any time in pregnancy. The reference was lamotrigine in the same exposure window. Results were obtained with linear regression analysis models and were adjusted for birth year, maternal age at delivery, education, country of origin, marital status, body mass index, smoking in current pregnancy, alcohol dependence, diabetes, hypertension, epilepsy, depression, bipolar disorder, migraine, chronic pain, and other psychiatric disorders.

1. Association between in-utero levetiracetam exposure and the endpoints duration of pregnancy and size at birth

|  | | **Difference (95% CI)** | | | | **Odds ratio** |
| --- | --- | --- | --- | --- | --- | --- |
|  | | | **At percentile** | | | **(95% CI)** |
|  | **Exposed to levetiracetam/ reference** | **Mean** | **10^th^** | **50^th^** | **90^th^** |  |
| **Pregnancy duration (days)                                          Preterm birth** | | | | | | |
| Use any time in pregnancy, levetiracetam vs. lamotrigine | 213 / 2,133 | -0.5 (-2.6 to 1.6) | -1.0 (-6.3 to 4.3) | 0.6 (-1.2 to 2.4) | 1.6 (-0.2 to 3.3) | 1.3 (0.8 to 2.3) |
| Use in first trimester, levetiracetam vs. lamotrigine | 184 / 1,938 | -0.7 (-2.9 to 1.5) | -1.7 (-7.3 to 4.0) | 0.3 (-1.8 to 2.5) | 1.8 (0.1 to 3.6) | 1.6 (0.9 to 2.8) |
| Continuers, levetiracetam vs. lamotrigine | 144 / 990 | -1.1 (-3.5 to 1.4) | -1.0 (-7.7 to 5.7) | 1.0 (-1.7 to 3.6) | 0.2 (-2.5 to 2.9) | 1.3 (0.6 to 2.6) |
| Mother with epilepsy, levetiracetam vs. lamotrigine | 209 / 1,451 | -0.5 (-2.5 to 1.6) | -0.2 (-5.1 to 4.7) | -0.2 (-2.2 to 1.8) | 1.3 (-0.5 to 3.2) | 1.3 (0.8 to 2.4) |
| Mother with chronic pain, levetiracetam vs. lamotrigine | 52 / 536 | 2.6 (-2.1 to 7.4) | 5.3 (-5.3 to 16.0) | 2.1 (-1.9 to 6.1) | 5.0 (-0.5 to 10.5) | Not applicable |
| Monotherapy, levetiracetam vs. lamotrigine | 126 / 1,787 | -0.5 (-3.1 to 2.0) | -0.6 (-7.5 to 6.3) | 0.3 (-2.2 to 2.9) | 1.4 (-1.2 to 3.9) | 1.4 (0.7 to 2.8) |
| Polytherapy, levetiracetam vs. lamotrigine | 87 / 346 | -0.1 (-4.0 to 3.8) | -0.5 (-8.4 to 7.4) | 0.3 (-3.7 to 4.2) | -1.1 (-4.4 to 2.3) | 1.0 (0.4 to 2.7) |
| Definite exposure, levetiracetam vs. lamotrigine | 121 / 1,045 | -2.3 (-4.9 to 0.3) | -2.0 (-10.4 to 6.5) | -1.0 (-4.2 to 2.2) | 2.2 (-1.2 to 5.5) | 1.5 (0.8 to 3.1) |
| Complete-case analysis, levetiracetam vs. lamotrigine | 197 / 1,917 | -0.7 (-2.8 to 1.5) | -0.5 (-5.3 to 4.3) | 0.0 (-1.9 to 1.9) | 1.3 (-0.7 to 3.3) | 1.3 (0.7 to 2.3) |
| Singletons with no MCMs, levetiracetam vs. lamotrigine | 190 / 1,923 | 0.4 (-1.5 to 2.4) | -0.5 (-4.5 to 3.5) | 0.2 (-1.6 to 2.1) | 1.2 (-0.7 to 3.1) | 1.1 (0.6 to 2.2) |
| First infant per woman, levetiracetam vs. lamotrigine | 152 / 1,627 | 0.7 (-1.8 to 3.2) | 0.2 (-7.0 to 7.4) | 1.9 (-0.3 to 4.2) | 1.3 (-0.7 to 3.2) | 1.1 (0.6 to 2.1) |
| Female infants, levetiracetam vs. lamotrigine | 93 / 1,114 | 0.7 (-2.3 to 3.8) | 1.8 (-3.8 to 7.4) | 0.4 (-3.5 to 4.2) | 2.5 (-0.4 to 5.4) | Not applicable |
| Male infants, levetiracetam vs. lamotrigine | 120 / 1,019 | -1.5 (-4.3 to 1.3) | -1.4 (-9.5 to 6.7) | 0.8 (-1.7 to 3.4) | 0.0 (-2.2 to 2.3) | 2.2 (1.1 to 4.3) |
| High vs. low dose of levetiracetam | 89 / 89 | -0.2 (-4.6 to 4.3) | -5.7 (-18.1 to 6.8) | -0.0 (-4.4 to 4.4) | 1.1 (-3.4 to 5.6) | 0.4 (0.1 to 1.6) |
| **Birth weight z-score                                                             SGA** | | | | | | |
| Use any time in pregnancy, levetiracetam vs. lamotrigine | 215 / 2,157 | -0.1 (-0.3 to 0.0) | -0.1 (-0.4 to 0.1) | 0.0 (-0.1 to 0.2) | -0.2 (-0.4 to 0.0) | 1.3 (0.5 to 3.0) |
| Use in first trimester, levetiracetam vs. lamotrigine | 186 / 1,961 | -0.1 (-0.3 to 0.0) | -0.2 (-0.5 to 0.0) | 0.0 (-0.1 to 0.2) | -0.1 (-0.3 to 0.1) | 1.8 (0.7 to 4.3) |
| Continuers, levetiracetam vs. lamotrigine | 146 / 998 | -0.1 (-0.3 to 0.1) | -0.2 (-0.6 to 0.2) | -0.0 (-0.2 to 0.1) | -0.2 (-0.5 to 0.1) | 1.7 (0.6 to 4.5) |
| Mother with epilepsy, levetiracetam vs. lamotrigine | 211 / 1,463 | -0.1 (-0.3 to 0.0) | -0.1 (-0.4 to 0.2) | 0.0 (-0.1 to 0.2) | -0.1 (-0.3 to 0.1) | 1.2 (0.5 to 2.9) |
| Mother with chronic pain, levetiracetam vs. lamotrigine | 51 / 546 | -0.1 (-0.4 to 0.3) | 0.2 (-0.8 to 1.3) | 0.2 (-0.2 to 0.5) | -0.4 (-0.9 to 0.1) | Not applicable |
| Monotherapy, levetiracetam vs. lamotrigine | 127 / 1,808 | 0.1 (-0.1 to 0.3) | 0.2 (-0.2 to 0.6) | 0.2 (0.0 to 0.3) | -0.1 (-0.4 to 0.2) | Not applicable |
| Polytherapy, levetiracetam vs. lamotrigine | 88 / 349 | -0.5 (-0.7 to -0.2) | -0.5 (-1.0 to 0.0) | -0.5 (-0.9 to -0.1) | -0.4 (-0.8 to 0.1) | Not applicable |
| Definite exposure, levetiracetam vs. lamotrigine | 123 / 1,055 | -0.0 (-0.2 to 0.2) | 0.1 (-0.3 to 0.5) | 0.0 (-0.2 to 0.2) | -0.1 (-0.4 to 0.1) | Not applicable |
| Complete-case analysis, levetiracetam vs. lamotrigine | 199 / 1,934 | -0.1 (-0.3 to 0.0) | -0.1 (-0.4 to 0.1) | 0.0 (-0.1 to 0.2) | -0.1 (-0.3 to 0.1) | 1.2 (0.5 to 2.9) |
| Singletons with no MCMs, levetiracetam vs. lamotrigine | 190 / 1,919 | -0.1 (-0.3 to 0.1) | -0.1 (-0.4 to 0.1) | 0.1 (-0.1 to 0.3) | -0.1 (-0.4 to 0.1) | 1.2 (0.5 to 3.0) |
| First infant per woman, levetiracetam vs. lamotrigine | 152 / 1,622 | -0.1 (-0.3 to 0.0) | -0.1 (-0.4 to 0.3) | -0.1 (-0.3 to 0.1) | -0.1 (-0.4 to 0.2) | 1.4 (0.5 to 3.5) |
| Female infants, levetiracetam vs. lamotrigine | 96 / 1,126 | -0.1 (-0.3 to 0.1) | -0.1 (-0.4 to 0.3) | 0.0 (-0.2 to 0.2) | -0.2 (-0.6 to 0.1) | Not applicable |
| Male infants, levetiracetam vs. lamotrigine | 119 / 1,031 | -0.1 (-0.3 to 0.1) | -0.3 (-0.7 to 0.1) | -0.1 (-0.3 to 0.2) | -0.1 (-0.4 to 0.2) | 1.9 (0.7 to 4.9) |
| High vs. low dose of levetiracetam | 90 / 91 | 0.1 (-0.1 to 0.4) | 0.2 (-0.7 to 1.2) | 0.1 (-0.2 to 0.4) | 0.4 (-0.1 to 0.9) | Not applicable |
| **Birth length z-score** | | | | | | |
| Use any time in pregnancy, levetiracetam vs. lamotrigine | 213 / 2,128 | -0.0 (-0.1 to 0.1) | -0.1 (-0.3 to 0.2) | 0.1 (-0.0 to 0.3) | -0.1 (-0.4 to 0.2) |  |
| Use in first trimester, levetiracetam vs. lamotrigine | 184 / 1,937 | -0.0 (-0.2 to 0.1) | -0.0 (-0.3 to 0.3) | 0.1 (-0.0 to 0.3) | -0.2 (-0.4 to 0.1) |  |
| Continuers, levetiracetam vs. lamotrigine | 144 / 983 | 0.1 (-0.1 to 0.2) | 0.2 (-0.2 to 0.5) | 0.1 (-0.0 to 0.3) | -0.2 (-0.4 to 0.1) |  |
| Mother with epilepsy, levetiracetam vs. lamotrigine | 209 / 1,444 | 0.0 (-0.1 to 0.2) | 0.0 (-0.2 to 0.3) | 0.1 (-0.0 to 0.2) | -0.1 (-0.3 to 0.2) |  |
| Mother with chronic pain, levetiracetam vs. lamotrigine | 51 / 536 | 0.1 (-0.2 to 0.4) | 0.4 (-0.3 to 1.1) | 0.3 (0.1 to 0.5) | 0.1 (-0.5 to 0.7) |  |
| Monotherapy, levetiracetam vs. lamotrigine | 125 / 1,788 | 0.2 (0.0 to 0.4) | 0.4 (0.1 to 0.7) | 0.2 (0.0 to 0.4) | 0.1 (-0.3 to 0.5) |  |
| Polytherapy, levetiracetam vs. lamotrigine | 88 / 340 | -0.2 (-0.5 to 0.0) | -0.5 (-0.9 to -0.1) | -0.0 (-0.3 to 0.3) | -0.3 (-0.7 to 0.2) |  |
| Definite exposure, levetiracetam vs. lamotrigine | 122 / 1,046 | 0.2 (-0.0 to 0.3) | 0.4 (0.0 to 0.7) | 0.2 (0.0 to 0.4) | -0.1 (-0.3 to 0.2) |  |
| Complete-case analysis, levetiracetam vs. lamotrigine | 199 / 1,906 | -0.0 (-0.2 to 0.1) | -0.1 (-0.4 to 0.2) | 0.1 (-0.0 to 0.3) | -0.1 (-0.4 to 0.2) |  |
| Singletons with no MCMs, levetiracetam vs. lamotrigine | 189 / 1,898 | 0.0 (-0.1 to 0.2) | 0.0 (-0.2 to 0.3) | 0.1 (-0.1 to 0.2) | -0.1 (-0.4 to 0.2) |  |
| First infant per woman, levetiracetam vs. lamotrigine | 151 / 1,600 | 0.0 (-0.1 to 0.2) | -0.0 (-0.4 to 0.3) | 0.1 (-0.1 to 0.3) | -0.0 (-0.5 to 0.4) |  |
| Female infants, levetiracetam vs. lamotrigine | 95 / 1,110 | -0.0 (-0.3 to 0.2) | -0.1 (-0.5 to 0.3) | 0.1 (-0.1 to 0.3) | -0.2 (-0.6 to 0.2) |  |
| Male infants, levetiracetam vs. lamotrigine | 118 / 1,018 | 0.1 (-0.1 to 0.2) | -0.1 (-0.5 to 0.3) | 0.0 (-0.1 to 0.2) | 0.2 (-0.2 to 0.5) |  |
| High vs. low dose of levetiracetam | 89 / 90 | 0.1 (-0.1 to 0.4) | 0.1 (-0.5 to 0.7) | 0.0 (-0.3 to 0.4) | 0.3 (-0.2 to 0.8) |  |
| **Birth head circumference z-score                                    Microcephaly** | | | | | | |
| Use any time in pregnancy, levetiracetam vs. lamotrigine | 206 / 2,103 | -0.1 (-0.3 to 0.1) | -0.1 (-0.4 to 0.2) | -0.1 (-0.3 to 0.1) | -0.1 (-0.4 to 0.1) | 1.4 (0.6 to 3.5) |
| Use in first trimester, levetiracetam vs. lamotrigine | 178 / 1,912 | -0.1 (-0.3 to 0.1) | 0.0 (-0.4 to 0.4) | -0.1 (-0.3 to 0.1) | -0.1 (-0.4 to 0.1) | 1.6 (0.6 to 4.4) |
| Continuers, levetiracetam vs. lamotrigine | 140 / 978 | -0.0 (-0.2 to 0.1) | 0.1 (-0.3 to 0.5) | -0.0 (-0.3 to 0.2) | -0.0 (-0.4 to 0.4) | Not applicable |
| Mother with epilepsy, levetiracetam vs. lamotrigine | 202 / 1,422 | -0.1 (-0.3 to 0.1) | -0.0 (-0.4 to 0.3) | -0.1 (-0.3 to 0.0) | -0.2 (-0.5 to 0.0) | 1.5 (0.6 to 3.9) |
| Mother with chronic pain, levetiracetam vs. lamotrigine | 50 / 536 | -0.0 (-0.3 to 0.3) | -0.2 (-0.9 to 0.6) | -0.1 (-0.3 to 0.2) | -0.3 (-0.9 to 0.2) | Not applicable |
| Monotherapy, levetiracetam vs. lamotrigine | 122 / 1,767 | 0.2 (0.0 to 0.4) | 0.3 (-0.0 to 0.7) | 0.1 (-0.1 to 0.4) | 0.1 (-0.3 to 0.4) | Not applicable |
| Polytherapy, levetiracetam vs. lamotrigine | 84 / 336 | -0.6 (-0.9 to -0.3) | -0.4 (-1.0 to 0.2) | -0.8 (-1.0 to -0.5) | -0.5 (-0.9 to -0.0) | 2.7 (0.7 to 9.6) |
| Definite exposure, levetiracetam vs. lamotrigine | 118 / 1,036 | -0.0 (-0.2 to 0.2) | 0.2 (-0.2 to 0.6) | -0.1 (-0.4 to 0.1) | -0.1 (-0.5 to 0.3) | Not applicable |
| Complete-case analysis, levetiracetam vs. lamotrigine | 192 / 1,888 | -0.1 (-0.3 to 0.1) | -0.0 (-0.4 to 0.3) | -0.1 (-0.3 to 0.1) | -0.1 (-0.4 to 0.1) | 1.6 (0.6 to 4.0) |
| Singletons with no MCMs, levetiracetam vs. lamotrigine | 183 / 1,877 | -0.1 (-0.2 to 0.1) | -0.1 (-0.3 to 0.2) | -0.1 (-0.3 to 0.2) | -0.0 (-0.3 to 0.3) | Not applicable |
| First infant per woman, levetiracetam vs. lamotrigine | 145 / 1,582 | -0.1 (-0.2 to 0.1) | -0.0 (-0.3 to 0.3) | -0.1 (-0.3 to 0.1) | -0.2 (-0.5 to 0.1) | Not applicable |
| Female infants, levetiracetam vs. lamotrigine | 91 / 1,095 | -0.2 (-0.4 to 0.0) | -0.3 (-0.7 to 0.1) | -0.1 (-0.3 to 0.2) | -0.3 (-0.7 to 0.1) | Not applicable |
| Male infants, levetiracetam vs. lamotrigine | 115 / 1,008 | 0.0 (-0.2 to 0.2) | 0.2 (-0.2 to 0.5) | -0.1 (-0.3 to 0.2) | -0.2 (-0.6 to 0.2) | Not applicable |
| High vs. low dose of levetiracetam | 87 / 89 | -0.0 (-0.3 to 0.3) | 0.4 (-0.2 to 1.0) | 0.1 (-0.3 to 0.4) | -0.4 (-1.0 to 0.2) | Not applicable |
| **Birth weight (grams)** | | | | | | |
| Use any time in pregnancy, levetiracetam vs. lamotrigine | 215 / 2,157 | -79 (-166 to 8) | -62 (-226 to 102) | -29 (-115 to 57) | -63 (-194 to 69) |  |
| Use in first trimester, levetiracetam vs. lamotrigine | 186 / 1,961 | -95 (-189 to -2) | -104 (-282 to 73) | -30 (-126 to 66) | -81 (-220 to 57) |  |
| Continuers, levetiracetam vs. lamotrigine | 146 / 998 | -104 (-208 to 0) | -57 (-262 to 148) | -31 (-121 to 60) | -43 (-195 to 110) |  |
| Mother with epilepsy, levetiracetam vs. lamotrigine | 211 / 1,463 | -74 (-162 to 14) | -61 (-198 to 76) | -33 (-120 to 54) | 15 (-133 to 163) |  |
| Mother with chronic pain, levetiracetam vs. lamotrigine | 51 / 546 | 57 (-139 to 252) | 170 (-233 to 574) | 53 (-141 to 246) | -144 (-396 to 107) |  |
| Monotherapy, levetiracetam vs. lamotrigine | 127 / 1,808 | 24 (-87 to 135) | 69 (-210 to 347) | 63 (-33 to 160) | 22 (-125 to 169) |  |
| Polytherapy, levetiracetam vs. lamotrigine | 88 / 349 | -207 (-357 to -56) | 34 (-347 to 415) | -207 (-376 to -39) | -165 (-348 to 18) |  |
| Definite exposure, levetiracetam vs. lamotrigine | 123 / 1,055 | -93 (-206 to 21) | -62 (-452 to 328) | -30 (-142 to 82) | -26 (-218 to 166) |  |
| Complete-case analysis, levetiracetam vs. lamotrigine | 199 / 1,934 | -84 (-175 to 7) | -80 (-234 to 74) | -33 (-126 to 61) | -10 (-166 to 145) |  |
| Singletons with no MCMs, levetiracetam vs. lamotrigine | 190 / 1,919 | -31 (-115 to 54) | -7 (-135 to 120) | -2 (-94 to 90) | -39 (-176 to 98) |  |
| First infant per woman, levetiracetam vs. lamotrigine | 152 / 1,622 | -41 (-140 to 58) | -1 (-149 to 146) | -35 (-141 to 72) | -17 (-181 to 147) |  |
| Female infants, levetiracetam vs. lamotrigine | 96 / 1,126 | -91 (-220 to 38) | -12 (-404 to 381) | -42 (-141 to 57) | -102 (-271 to 68) |  |
| Male infants, levetiracetam vs. lamotrigine | 119 / 1,031 | -82 (-200 to 36) | -119 (-351 to 114) | 0 (-128 to 128) | -47 (-202 to 109) |  |
| High vs. low dose of levetiracetam | 90 / 91 | 42 (-131 to 214) | -124 (-591 to 343) | 80 (-109 to 268) | 119 (-145 to 383) |  |
| **Birth length (cm)** | | | | | | |
| Use any time in pregnancy, levetiracetam vs. lamotrigine | 213 / 2,128 | -0.2 (-0.6 to 0.2) | -0.0 (-1.1 to 1.1) | -0.0 (-0.2 to 0.2) | 0.0 (-0.5 to 0.5) |  |
| Use in first trimester, levetiracetam vs. lamotrigine | 184 / 1,937 | -0.2 (-0.7 to 0.2) | -0.1 (-1.1 to 1.0) | -0.0 (-0.3 to 0.3) | -0.0 (-0.5 to 0.5) |  |
| Continuers, levetiracetam vs. lamotrigine | 144 / 983 | -0.2 (-0.7 to 0.2) | -0.0 (-1.3 to 1.3) | 0.3 (-0.1 to 0.7) | -0.2 (-0.7 to 0.3) |  |
| Mother with epilepsy, levetiracetam vs. lamotrigine | 209 / 1,444 | -0.1 (-0.5 to 0.2) | 0.0 (-1.0 to 1.0) | 0.0 (-0.3 to 0.3) | 0.0 (-0.4 to 0.4) |  |
| Mother with chronic pain, levetiracetam vs. lamotrigine | 51 / 536 | 0.7 (-0.2 to 1.6) | 1.3 (-0.3 to 2.8) | 0.7 (0.1 to 1.3) | 0.5 (-0.7 to 1.7) |  |
| Monotherapy, levetiracetam vs. lamotrigine | 125 / 1,788 | 0.2 (-0.3 to 0.7) | 0.0 (-0.9 to 0.9) | 1.0 (0.4 to 1.6) | 0.3 (-0.3 to 0.9) |  |
| Polytherapy, levetiracetam vs. lamotrigine | 88 / 340 | -0.6 (-1.2 to 0.1) | -0.7 (-2.9 to 1.5) | -0.5 (-1.0 to 0.1) | -0.6 (-1.4 to 0.2) |  |
| Definite exposure, levetiracetam vs. lamotrigine | 122 / 1,046 | -0.2 (-0.7 to 0.3) | -0.1 (-2.0 to 1.8) | 0.0 (-0.4 to 0.5) | 0.0 (-0.6 to 0.6) |  |
| Complete-case analysis, levetiracetam vs. lamotrigine | 199 / 1,906 | -0.3 (-0.7 to 0.1) | -0.0 (-1.2 to 1.2) | -0.0 (-0.2 to 0.2) | -0.0 (-0.4 to 0.4) |  |
| Singletons with no MCMs, levetiracetam vs. lamotrigine | 189 / 1,898 | 0.1 (-0.3 to 0.5) | 0.0 (-0.6 to 0.7) | -0.0 (-0.3 to 0.3) | -0.0 (-0.5 to 0.5) |  |
| First infant per woman, levetiracetam vs. lamotrigine | 151 / 1,600 | 0.1 (-0.3 to 0.6) | 0.0 (-1.0 to 1.0) | 0.0 (-0.4 to 0.4) | 0.3 (-0.4 to 1.0) |  |
| Female infants, levetiracetam vs. lamotrigine | 95 / 1,110 | -0.4 (-1.0 to 0.2) | -0.0 (-2.0 to 1.9) | 0.0 (-0.3 to 0.3) | -0.5 (-1.4 to 0.4) |  |
| Male infants, levetiracetam vs. lamotrigine | 118 / 1,018 | -0.1 (-0.6 to 0.5) | 0.1 (-1.3 to 1.5) | 0.1 (-0.4 to 0.7) | -0.1 (-0.8 to 0.6) |  |
| High vs. low dose of levetiracetam | 89 / 90 | 0.0 (-0.8 to 0.9) | -1.7 (-4.2 to 0.7) | 0.1 (-0.5 to 0.8) | -0.1 (-1.6 to 1.3) |  |
| **Birth head circumference (cm)** | | | | | | |
| Use any time in pregnancy, levetiracetam vs. lamotrigine | 206 / 2,103 | -0.2 (-0.5 to 0.0) | -0.0 (-0.5 to 0.5) | -0.0 (-0.0 to 0.0) | 0.0 (-0.3 to 0.3) |  |
| Use in first trimester, levetiracetam vs. lamotrigine | 178 / 1,912 | -0.3 (-0.6 to -0.0) | -0.0 (-0.7 to 0.7) | -0.0 (-0.0 to 0.0) | 0.0 (-0.3 to 0.3) |  |
| Continuers, levetiracetam vs. lamotrigine | 140 / 978 | -0.3 (-0.6 to 0.0) | -0.4 (-1.0 to 0.3) | -0.0 (-0.3 to 0.3) | 0.0 (-0.5 to 0.5) |  |
| Mother with epilepsy, levetiracetam vs. lamotrigine | 202 / 1,422 | -0.3 (-0.5 to 0.0) | -0.0 (-0.5 to 0.5) | -0.0 (-0.0 to 0.0) | -0.0 (-0.3 to 0.3) |  |
| Mother with chronic pain, levetiracetam vs. lamotrigine | 50 / 536 | 0.3 (-0.3 to 0.9) | 0.5 (-0.5 to 1.5) | -0.0 (-0.6 to 0.6) | -0.2 (-1.3 to 0.9) |  |
| Monotherapy, levetiracetam vs. lamotrigine | 122 / 1,767 | 0.1 (-0.2 to 0.5) | 0.2 (-0.5 to 0.8) | 0.0 (-0.3 to 0.3) | 0.2 (-0.2 to 0.5) |  |
| Polytherapy, levetiracetam vs. lamotrigine | 84 / 336 | -0.9 (-1.3 to -0.4) | -0.6 (-1.9 to 0.8) | -0.8 (-1.3 to -0.3) | -0.3 (-1.3 to 0.6) |  |
| Definite exposure, levetiracetam vs. lamotrigine | 118 / 1,036 | -0.3 (-0.7 to 0.0) | -0.0 (-1.0 to 1.0) | -0.0 (-0.2 to 0.2) | 0.0 (-0.4 to 0.4) |  |
| Complete-case analysis, levetiracetam vs. lamotrigine | 192 / 1,888 | -0.3 (-0.6 to -0.0) | -0.2 (-0.8 to 0.5) | -0.0 (-0.0 to 0.0) | 0.0 (-0.3 to 0.3) |  |
| Singletons with no MCMs, levetiracetam vs. lamotrigine | 183 / 1,877 | -0.0 (-0.3 to 0.2) | -0.0 (-0.4 to 0.4) | 0.0 (-0.0 to 0.0) | 0.1 (-0.2 to 0.4) |  |
| First infant per woman, levetiracetam vs. lamotrigine | 145 / 1,582 | -0.0 (-0.4 to 0.3) | 0.1 (-0.5 to 0.7) | -0.0 (-0.1 to 0.1) | 0.1 (-0.3 to 0.4) |  |
| Female infants, levetiracetam vs. lamotrigine | 91 / 1,095 | -0.5 (-0.9 to -0.1) | -0.6 (-1.9 to 0.7) | -0.0 (-0.6 to 0.5) | -0.1 (-0.5 to 0.4) |  |
| Male infants, levetiracetam vs. lamotrigine | 115 / 1,008 | -0.1 (-0.5 to 0.2) | -0.0 (-0.6 to 0.6) | -0.2 (-0.7 to 0.2) | 0.0 (-0.4 to 0.5) |  |
| High vs. low dose of levetiracetam | 87 / 89 | -0.1 (-0.7 to 0.5) | 0.4 (-1.3 to 2.2) | -0.0 (-0.5 to 0.5) | -0.3 (-1.1 to 0.6) |  |

AED = antiepileptic drug; CI = confidence interval; MCM = major congenital malformation; SGA = small for gestational age

AED use was ascertained at any time in pregnancy, except where noted (indented rows). Analyses on continuers used data from deliveries in 2006-2013. In analyses of carbamazepine vs. lamotrigine, the reference was lamotrigine in the same exposure window. In dose-response analyses, the reference was the bottom tertile of mean daily dose of carbamazepine (2006-2013). To facilitate convergence of quantile regression models for birth length and head circumference in cm, we implemented 1% jittering of birth length and head circumference by introducing random noise with uniform distribution and width 0.01 around the observation´s value. All results were adjusted for birth year, maternal age at delivery, education, country of origin, marital status, body mass index, smoking in current pregnancy, alcohol dependence, diabetes, hypertension, epilepsy, depression, bipolar disorder, migraine, chronic pain, and other psychiatric disorders. When the smallest cell count was < 5, we did not produce adjusted results ("not applicable"). Models restricted to polytherapy compared infants exposed to carbamazepine and another AED (except lamotrigine) with those exposed to lamotrigine and another AED (except carbamazepine).

1. Effect-measure modification analysis for the association between in-utero levetiracetam exposure and the endpoints duration of pregnancy and size at birth

|  | | | **Adjusted difference (95% CI)** | | |  |
| --- | --- | --- | --- | --- | --- | --- |
| **Potential EMM** | **Endpoint** | **Exposed to levetiracetam / lamotrigine** | **Levetiracetam** | **Potential EMM** | **Interaction term** | **P-value for interaction term** |
| Smoking in pregnancy | Pregnancy duration (days) | 213 / 2,133 | -0.8 (-3.0 to 1.3) | -1.4 (-3.2 to 0.3) | 4.4 (-3.0 to 11.7) | 0.248 |
|  | Birth weight z-score | 215 / 2,157 | -0.1 (-0.3 to 0.1) | -0.4 (-0.6 to -0.3) | -0.4 (-0.9 to 0.2) | 0.193 |
|  | Birth length z-score | 213 / 2,128 | -0.0 (-0.1 to 0.1) | -0.5 (-0.6 to -0.3) | 0.0 (-0.5 to 0.5) | 0.969 |
|  | Head circumference z-score | 206 / 2,103 | -0.1 (-0.2 to 0.1) | -0.3 (-0.5 to -0.2) | -0.4 (-1.0 to 0.1) | 0.129 |
|  | Birth weight (grams) | 215 / 2,157 | -80 (-170 to 11) | -193 (-266 to -120) | 11 (-303 to 325) | 0.945 |
|  | Birth length (cm) | 213 / 2,128 | -0.2 (-0.6 to 0.2) | -0.9 (-1.2 to -0.6) | 0.8 (-0.6 to 2.2) | 0.274 |
|  | Head circumference (cm) | 206 / 2,103 | -0.2 (-0.5 to 0.0) | -0.4 (-0.6 to -0.2) | -0.1 (-1.1 to 0.8) | 0.808 |
| Use of SSRIs in pregnancy | Pregnancy duration (days) | 213 / 2,133 | -0.1 (-2.2 to 2.0) | -1.6 (-3.5 to 0.3) | -11.5 (-22.3 to -0.6) | 0.038 |
|  | Birth weight z-score | 215 / 2,157 | -0.1 (-0.3 to 0.0) | -0.1 (-0.2 to 0.1) | 0.2 (-0.6 to 1.0) | 0.635 |
|  | Birth length z-score | 213 / 2,128 | -0.0 (-0.2 to 0.1) | -0.3 (-0.4 to -0.1) | 0.2 (-0.5 to 1.0) | 0.493 |
|  | Head circumference z-score | 206 / 2,103 | -0.1 (-0.3 to 0.1) | -0.1 (-0.2 to 0.1) | 0.1 (-0.7 to 0.8) | 0.844 |
|  | Birth weight (grams) | 215 / 2,157 | -60 (-149 to 29) | -44 (-125 to 38) | -495 (-928 to -62) | 0.025 |
|  | Birth length (cm) | 213 / 2,128 | -0.1 (-0.5 to 0.3) | -0.5 (-0.9 to -0.2) | -2.5 (-4.4 to -0.6) | 0.011 |
|  | Head circumference (cm) | 206 / 2,103 | -0.2 (-0.4 to 0.1) | -0.1 (-0.3 to 0.2) | -2.0 (-3.3 to -0.7) | 0.003 |

AED = antiepileptic drug; CI = confidence interval; EMM = effect-measure modifier; SSRI = selective serotonin reuptake inhibitor.

AED use was ascertained at any time in pregnancy. The reference was lamotrigine in the same exposure window. Results were obtained with linear regression analysis models and were adjusted for birth year, maternal age at delivery, education, country of origin, marital status, body mass index, smoking in current pregnancy, alcohol dependence, diabetes, hypertension, epilepsy, depression, bipolar disorder, migraine, chronic pain, and other psychiatric disorders.

1. Association between in-utero valproic acid exposure and the endpoints duration of pregnancy and size at birth

|  | | **Difference (95% CI)** | | | | **Odds ratio** |
| --- | --- | --- | --- | --- | --- | --- |
|  | | | **At percentile** | | | **(95% CI)** |
|  | **Exposed to valproic acid/ reference** | **Mean** | **10^th^** | **50^th^** | **90^th^** |  |
| **Pregnancy duration (days)                                                     Preterm birth** | | | | | | |
| Use any time in pregnancy, valproic acid vs. lamotrigine | 985 / 2,086 | -0.0 (-1.2 to 1.2) | -1.9 (-5.3 to 1.4) | 1.0 (-0.3 to 2.3) | 1.6 (0.4 to 2.8) | 1.5 (1.1 to 2.0) |
| Use in first trimester, valproic acid vs. lamotrigine | 845 / 1,902 | -0.1 (-1.3 to 1.2) | -1.3 (-4.9 to 2.2) | 0.8 (-0.4 to 2.0) | 1.4 (0.3 to 2.5) | 1.6 (1.1 to 2.2) |
| Continuers, valproic acid vs. lamotrigine | 253 / 996 | -0.0 (-2.0 to 2.0) | -3.9 (-10.6 to 2.7) | 1.8 (-0.4 to 3.9) | 2.4 (0.7 to 4.1) | 1.7 (1.1 to 2.8) |
| Mother with epilepsy, valproic acid vs. lamotrigine | 805 / 1,421 | -0.2 (-1.5 to 1.2) | -4.0 (-7.8 to -0.2) | 1.0 (-0.2 to 2.2) | 1.4 (0.3 to 2.4) | 1.7 (1.2 to 2.4) |
| Mother with chronic pain, valproic acid vs. lamotrigine | 144 / 537 | -0.3 (-3.3 to 2.7) | -0.0 (-7.0 to 7.0) | -1.1 (-4.6 to 2.4) | -0.0 (-3.2 to 3.2) | 1.3 (0.7 to 2.4) |
| Monotherapy, valproic acid vs. lamotrigine | 870 / 1,787 | 0.3 (-1.0 to 1.6) | 0.3 (-2.8 to 3.3) | 1.3 (-0.1 to 2.8) | 1.6 (0.4 to 2.8) | 1.3 (0.9 to 1.8) |
| Polytherapy, valproic acid vs. lamotrigine | 115 / 299 | -3.4 (-6.9 to 0.2) | -10.0 (-19.5 to -0.5) | 0.1 (-3.4 to 3.5) | 2.2 (-1.1 to 5.4) | 3.0 (1.5 to 6.2) |
| Definite exposure, valproic acid vs. lamotrigine | 265 / 1,048 | -0.5 (-2.4 to 1.5) | -5.5 (-12.5 to 1.6) | 1.4 (-0.7 to 3.4) | 1.2 (-0.6 to 3.1) | 1.6 (1.0 to 2.6) |
| Complete-case analysis, valproic acid vs. lamotrigine | 861 / 1,877 | -0.4 (-1.6 to 0.9) | -2.1 (-5.6 to 1.3) | 0.5 (-0.9 to 1.9) | 0.8 (-0.3 to 1.9) | 1.5 (1.1 to 2.0) |
| Singletons with no MCMs, valproic acid vs. lamotrigine | 813 / 1,891 | 0.5 (-0.6 to 1.7) | -0.4 (-3.1 to 2.3) | 1.1 (-0.2 to 2.4) | 1.6 (0.5 to 2.8) | 1.4 (1.0 to 2.0) |
| First infant per woman, valproic acid vs. lamotrigine | 686 / 1,600 | 0.4 (-1.1 to 1.9) | -3.7 (-7.8 to 0.3) | 1.4 (-0.2 to 2.9) | 1.6 (0.4 to 2.8) | 1.5 (1.0 to 2.1) |
| Female infants, valproic acid vs. lamotrigine | 480 / 1,094 | 0.6 (-1.1 to 2.4) | 1.6 (-2.3 to 5.5) | 1.1 (-0.6 to 2.9) | 1.7 (0.2 to 3.3) | 1.1 (0.7 to 1.8) |
| Male infants, valproic acid vs. lamotrigine | 505 / 992 | -0.7 (-2.4 to 1.0) | -3.8 (-7.6 to -0.0) | -0.1 (-1.6 to 1.5) | 0.9 (-0.6 to 2.4) | 1.9 (1.2 to 2.9) |
| High vs. low dose of valproic acid | 165 / 167 | -1.0 (-4.9 to 2.9) | -2.4 (-10.1 to 5.3) | -0.5 (-3.9 to 3.0) | 1.0 (-1.4 to 3.5) | 1.4 (0.5 to 3.4) |
| **Birth weight z-score                                                           SGA** | | | | | | |
| Use any time in pregnancy, valproic acid vs. lamotrigine | 992 / 2,110 | -0.0 (-0.1 to 0.0) | -0.1 (-0.3 to 0.1) | -0.1 (-0.2 to 0.1) | 0.1 (-0.1 to 0.2) | 1.9 (1.2 to 2.9) |
| Use in first trimester, valproic acid vs. lamotrigine | 852 / 1,924 | -0.1 (-0.2 to 0.0) | -0.1 (-0.3 to 0.1) | -0.0 (-0.2 to 0.1) | 0.1 (-0.1 to 0.2) | 2.4 (1.5 to 3.8) |
| Continuers, valproic acid vs. lamotrigine | 257 / 1,004 | -0.1 (-0.3 to 0.1) | -0.3 (-0.6 to 0.1) | -0.0 (-0.2 to 0.2) | 0.3 (0.0 to 0.5) | 2.5 (1.3 to 5.0) |
| Mother with epilepsy, valproic acid vs. lamotrigine | 811 / 1,433 | -0.1 (-0.2 to 0.0) | -0.1 (-0.3 to 0.1) | -0.1 (-0.2 to 0.1) | 0.1 (-0.1 to 0.2) | 1.7 (1.1 to 2.8) |
| Mother with chronic pain, valproic acid vs. lamotrigine | 145 / 547 | -0.1 (-0.3 to 0.2) | -0.1 (-0.5 to 0.3) | -0.0 (-0.3 to 0.2) | 0.4 (-0.0 to 0.8) | 3.0 (1.2 to 7.5) |
| Monotherapy, valproic acid vs. lamotrigine | 876 / 1,808 | -0.0 (-0.1 to 0.1) | -0.1 (-0.3 to 0.1) | -0.0 (-0.1 to 0.1) | 0.1 (-0.1 to 0.3) | 1.9 (1.2 to 3.2) |
| Polytherapy, valproic acid vs. lamotrigine | 116 / 302 | -0.2 (-0.5 to 0.0) | -0.2 (-0.6 to 0.2) | -0.1 (-0.4 to 0.1) | -0.4 (-0.7 to -0.1) | 2.6 (0.6 to 11.0) |
| Definite exposure, valproic acid vs. lamotrigine | 269 / 1,059 | -0.1 (-0.2 to 0.1) | -0.3 (-0.6 to 0.0) | -0.0 (-0.2 to 0.1) | 0.2 (-0.1 to 0.5) | 1.8 (0.9 to 3.8) |
| Complete-case analysis, valproic acid vs. lamotrigine | 866 / 1,896 | -0.0 (-0.1 to 0.1) | -0.1 (-0.2 to 0.1) | -0.0 (-0.1 to 0.1) | 0.1 (-0.1 to 0.2) | 1.6 (1.0 to 2.5) |
| Singletons with no MCMs, valproic acid vs. lamotrigine | 808 / 1,887 | -0.0 (-0.1 to 0.1) | -0.1 (-0.3 to 0.1) | -0.0 (-0.1 to 0.1) | 0.1 (-0.1 to 0.2) | 1.7 (1.0 to 2.7) |
| First infant per woman, valproic acid vs. lamotrigine | 682 / 1,596 | -0.1 (-0.2 to -0.0) | -0.3 (-0.5 to -0.1) | -0.1 (-0.2 to 0.0) | 0.0 (-0.2 to 0.2) | 2.3 (1.4 to 3.8) |
| Female infants, valproic acid vs. lamotrigine | 484 / 1,106 | -0.1 (-0.2 to 0.0) | -0.0 (-0.3 to 0.2) | -0.1 (-0.2 to 0.0) | 0.0 (-0.2 to 0.2) | 2.5 (1.3 to 5.0) |
| Male infants, valproic acid vs. lamotrigine | 508 / 1,004 | 0.0 (-0.1 to 0.1) | -0.1 (-0.3 to 0.1) | 0.0 (-0.1 to 0.2) | 0.2 (-0.0 to 0.4) | 1.5 (0.8 to 2.9) |
| High vs. low dose of valproic acid | 169 / 168 | -0.1 (-0.3 to 0.2) | -0.4 (-0.9 to 0.1) | -0.1 (-0.4 to 0.2) | 0.4 (-0.0 to 0.8) | Not applicable |
| **Birth length z-score** | | | | | | |
| Use any time in pregnancy, valproic acid vs. lamotrigine | 966 / 2,083 | 0.1 (0.0 to 0.2) | 0.0 (-0.1 to 0.2) | 0.1 (-0.0 to 0.2) | 0.2 (0.1 to 0.3) |  |
| Use in first trimester, valproic acid vs. lamotrigine | 828 / 1,901 | 0.1 (-0.0 to 0.2) | 0.0 (-0.1 to 0.2) | 0.1 (-0.0 to 0.2) | 0.2 (0.1 to 0.4) |  |
| Continuers, valproic acid vs. lamotrigine | 254 / 989 | 0.1 (-0.0 to 0.3) | 0.0 (-0.3 to 0.4) | 0.2 (-0.0 to 0.4) | 0.3 (0.0 to 0.5) |  |
| Mother with epilepsy, valproic acid vs. lamotrigine | 788 / 1,416 | 0.1 (0.0 to 0.2) | 0.0 (-0.2 to 0.2) | 0.1 (0.0 to 0.2) | 0.2 (0.1 to 0.4) |  |
| Mother with chronic pain, valproic acid vs. lamotrigine | 142 / 537 | -0.2 (-0.4 to 0.1) | -0.2 (-0.5 to 0.2) | -0.1 (-0.4 to 0.1) | 0.3 (-0.1 to 0.7) |  |
| Monotherapy, valproic acid vs. lamotrigine | 854 / 1,788 | 0.1 (-0.0 to 0.2) | -0.0 (-0.2 to 0.2) | 0.1 (-0.0 to 0.2) | 0.3 (0.1 to 0.4) |  |
| Polytherapy, valproic acid vs. lamotrigine | 112 / 295 | 0.0 (-0.2 to 0.3) | 0.2 (-0.2 to 0.5) | 0.2 (0.0 to 0.4) | -0.1 (-0.5 to 0.3) |  |
| Definite exposure, valproic acid vs. lamotrigine | 266 / 1,050 | 0.0 (-0.1 to 0.2) | -0.1 (-0.4 to 0.2) | 0.1 (-0.1 to 0.3) | 0.2 (-0.0 to 0.4) |  |
| Complete-case analysis, valproic acid vs. lamotrigine | 847 / 1,870 | 0.1 (0.0 to 0.2) | 0.1 (-0.1 to 0.3) | 0.1 (-0.0 to 0.2) | 0.2 (0.0 to 0.3) |  |
| Singletons with no MCMs, valproic acid vs. lamotrigine | 795 / 1,868 | 0.1 (0.0 to 0.2) | 0.1 (-0.1 to 0.3) | 0.1 (0.0 to 0.2) | 0.2 (-0.0 to 0.3) |  |
| First infant per woman, valproic acid vs. lamotrigine | 660 / 1,576 | 0.0 (-0.1 to 0.2) | -0.1 (-0.2 to 0.1) | 0.0 (-0.1 to 0.2) | 0.2 (-0.0 to 0.3) |  |
| Female infants, valproic acid vs. lamotrigine | 472 / 1,091 | 0.0 (-0.1 to 0.2) | 0.1 (-0.1 to 0.3) | 0.0 (-0.1 to 0.2) | -0.0 (-0.2 to 0.2) |  |
| Male infants, valproic acid vs. lamotrigine | 494 / 992 | 0.1 (0.0 to 0.3) | 0.0 (-0.2 to 0.2) | 0.2 (0.1 to 0.4) | 0.2 (0.0 to 0.4) |  |
| High vs. low dose of valproic acid | 167 / 167 | 0.2 (-0.1 to 0.4) | 0.3 (-0.2 to 0.8) | 0.1 (-0.1 to 0.4) | 0.3 (-0.2 to 0.8) |  |
| **Birth head circumference z-score                                        Microcephaly** | | | | | | |
| Use any time in pregnancy, valproic acid vs. lamotrigine | 931 / 2,059 | -0.2 (-0.2 to -0.1) | -0.1 (-0.3 to 0.0) | -0.1 (-0.2 to -0.1) | -0.2 (-0.3 to -0.0) | 1.7 (1.0 to 2.8) |
| Use in first trimester, valproic acid vs. lamotrigine | 802 / 1,877 | -0.1 (-0.2 to -0.0) | -0.1 (-0.2 to 0.1) | -0.2 (-0.3 to -0.1) | -0.2 (-0.3 to -0.0) | 1.8 (1.0 to 3.1) |
| Continuers, valproic acid vs. lamotrigine | 252 / 983 | -0.2 (-0.3 to -0.0) | -0.2 (-0.5 to 0.1) | -0.2 (-0.3 to -0.0) | -0.2 (-0.4 to 0.1) | 3.9 (1.7 to 9.0) |
| Mother with epilepsy, valproic acid vs. lamotrigine | 759 / 1,395 | -0.2 (-0.2 to -0.1) | -0.2 (-0.4 to -0.0) | -0.2 (-0.3 to -0.1) | -0.2 (-0.4 to -0.1) | 1.8 (1.1 to 3.2) |
| Mother with chronic pain, valproic acid vs. lamotrigine | 140 / 537 | -0.1 (-0.3 to 0.1) | -0.1 (-0.4 to 0.3) | -0.1 (-0.4 to 0.1) | 0.1 (-0.2 to 0.5) | 3.3 (0.9 to 11.8) |
| Monotherapy, valproic acid vs. lamotrigine | 824 / 1,767 | -0.1 (-0.2 to -0.0) | -0.1 (-0.2 to 0.1) | -0.1 (-0.2 to -0.0) | -0.1 (-0.3 to 0.0) | 1.8 (1.0 to 3.2) |
| Polytherapy, valproic acid vs. lamotrigine | 107 / 292 | -0.5 (-0.7 to -0.2) | -0.5 (-0.9 to -0.1) | -0.4 (-0.6 to -0.1) | -0.4 (-0.9 to 0.0) | 3.1 (1.0 to 9.8) |
| Definite exposure, valproic acid vs. lamotrigine | 266 / 1,040 | -0.2 (-0.3 to -0.0) | -0.1 (-0.4 to 0.2) | -0.1 (-0.3 to 0.1) | -0.3 (-0.6 to -0.0) | 2.1 (1.0 to 4.8) |
| Complete-case analysis, valproic acid vs. lamotrigine | 821 / 1,853 | -0.2 (-0.3 to -0.1) | -0.1 (-0.3 to 0.0) | -0.2 (-0.3 to -0.1) | -0.2 (-0.4 to -0.1) | 1.8 (1.1 to 3.1) |
| Singletons with no MCMs, valproic acid vs. lamotrigine | 770 / 1,848 | -0.1 (-0.2 to -0.0) | -0.1 (-0.3 to 0.0) | -0.1 (-0.2 to -0.0) | -0.2 (-0.3 to -0.0) | 1.5 (0.8 to 2.5) |
| First infant per woman, valproic acid vs. lamotrigine | 634 / 1,559 | -0.2 (-0.3 to -0.1) | -0.2 (-0.4 to 0.0) | -0.2 (-0.3 to -0.1) | -0.2 (-0.4 to -0.1) | 1.7 (1.0 to 3.0) |
| Female infants, valproic acid vs. lamotrigine | 458 / 1,078 | -0.2 (-0.3 to -0.0) | -0.1 (-0.3 to 0.2) | -0.2 (-0.3 to -0.0) | -0.2 (-0.3 to 0.0) | 1.8 (0.8 to 3.7) |
| Male infants, valproic acid vs. lamotrigine | 473 / 981 | -0.1 (-0.3 to -0.0) | -0.1 (-0.3 to 0.1) | -0.1 (-0.2 to 0.0) | -0.2 (-0.4 to 0.0) | 1.8 (0.9 to 3.6) |
| High vs. low dose of valproic acid | 166 / 162 | -0.2 (-0.5 to 0.0) | -0.4 (-1.0 to 0.2) | -0.1 (-0.4 to 0.2) | 0.1 (-0.3 to 0.6) | Not applicable |
| **Birth weight (grams)** | | | | | | |
| Use any time in pregnancy, valproic acid vs. lamotrigine | 992 / 2,110 | -27 (-79 to 24) | -68 (-157 to 22) | -18 (-66 to 31) | 45 (-40 to 131) |  |
| Use in first trimester, valproic acid vs. lamotrigine | 852 / 1,924 | -40 (-95 to 14) | -95 (-198 to 7) | -34 (-91 to 23) | 31 (-63 to 125) |  |
| Continuers, valproic acid vs. lamotrigine | 257 / 1,004 | -73 (-160 to 14) | -151 (-320 to 17) | -58 (-169 to 53) | 120 (-15 to 255) |  |
| Mother with epilepsy, valproic acid vs. lamotrigine | 811 / 1,433 | -42 (-99 to 15) | -113 (-222 to -3) | -11 (-71 to 49) | 50 (-35 to 134) |  |
| Mother with chronic pain, valproic acid vs. lamotrigine | 145 / 547 | -2 (-127 to 124) | -58 (-298 to 182) | -15 (-160 to 129) | 77 (-125 to 278) |  |
| Monotherapy, valproic acid vs. lamotrigine | 876 / 1,808 | -9 (-65 to 47) | -47 (-150 to 57) | -9 (-68 to 49) | 40 (-55 to 135) |  |
| Polytherapy, valproic acid vs. lamotrigine | 116 / 302 | -209 (-342 to -76) | -211 (-555 to 133) | -116 (-268 to 37) | -128 (-290 to 33) |  |
| Definite exposure, valproic acid vs. lamotrigine | 269 / 1,059 | -77 (-162 to 8) | -60 (-228 to 109) | -61 (-171 to 48) | 71 (-86 to 228) |  |
| Complete-case analysis, valproic acid vs. lamotrigine | 866 / 1,896 | -29 (-83 to 24) | -46 (-147 to 56) | -22 (-78 to 34) | 30 (-57 to 117) |  |
| Singletons with no MCMs, valproic acid vs. lamotrigine | 808 / 1,887 | 0 (-50 to 50) | 3 (-97 to 102) | 11 (-42 to 65) | 64 (-25 to 154) |  |
| First infant per woman, valproic acid vs. lamotrigine | 682 / 1,596 | -43 (-103 to 17) | -119 (-225 to -12) | -42 (-109 to 25) | 35 (-61 to 130) |  |
| Female infants, valproic acid vs. lamotrigine | 484 / 1,106 | -41 (-113 to 30) | -43 (-185 to 100) | -37 (-100 to 26) | -28 (-116 to 59) |  |
| Male infants, valproic acid vs. lamotrigine | 508 / 1,004 | -24 (-97 to 49) | -60 (-195 to 75) | 6 (-71 to 82) | 51 (-75 to 176) |  |
| High vs. low dose of valproic acid | 169 / 168 | -82 (-242 to 78) | -230 (-555 to 96) | -18 (-161 to 124) | 131 (-108 to 371) |  |
| **Birth length (cm)** | | | | | | |
| Use any time in pregnancy, valproic acid vs. lamotrigine | 966 / 2,083 | 0.1 (-0.1 to 0.4) | -0.5 (-1.0 to -0.0) | 0.0 (-0.3 to 0.3) | 0.5 (0.2 to 0.8) |  |
| Use in first trimester, valproic acid vs. lamotrigine | 828 / 1,901 | 0.1 (-0.1 to 0.3) | -0.6 (-1.0 to -0.1) | 0.3 (0.1 to 0.5) | 0.5 (0.1 to 0.8) |  |
| Continuers, valproic acid vs. lamotrigine | 254 / 989 | -0.0 (-0.4 to 0.4) | -0.8 (-1.7 to 0.0) | 0.3 (-0.1 to 0.7) | 1.0 (0.4 to 1.6) |  |
| Mother with epilepsy, valproic acid vs. lamotrigine | 788 / 1,416 | 0.1 (-0.2 to 0.4) | -0.7 (-1.2 to -0.2) | 0.3 (0.0 to 0.6) | 0.6 (0.2 to 0.9) |  |
| Mother with chronic pain, valproic acid vs. lamotrigine | 142 / 537 | -0.2 (-0.7 to 0.4) | -0.7 (-1.8 to 0.4) | -0.1 (-0.7 to 0.6) | 1.0 (0.0 to 2.0) |  |
| Monotherapy, valproic acid vs. lamotrigine | 854 / 1,788 | 0.2 (-0.1 to 0.4) | -0.4 (-0.8 to 0.0) | 0.3 (0.0 to 0.6) | 0.6 (0.2 to 0.9) |  |
| Polytherapy, valproic acid vs. lamotrigine | 112 / 295 | -0.5 (-1.1 to 0.1) | -1.4 (-3.1 to 0.4) | 0.1 (-0.5 to 0.7) | 0.1 (-0.8 to 0.9) |  |
| Definite exposure, valproic acid vs. lamotrigine | 266 / 1,050 | -0.2 (-0.6 to 0.2) | -1.0 (-1.8 to -0.2) | 0.2 (-0.2 to 0.6) | 0.4 (-0.2 to 1.0) |  |
| Complete-case analysis, valproic acid vs. lamotrigine | 847 / 1,870 | 0.1 (-0.1 to 0.3) | -0.4 (-0.9 to -0.0) | 0.0 (-0.2 to 0.3) | 0.4 (0.1 to 0.7) |  |
| Singletons with no MCMs, valproic acid vs. lamotrigine | 795 / 1,868 | 0.2 (0.0 to 0.5) | -0.0 (-0.4 to 0.4) | 0.0 (-0.3 to 0.3) | 0.4 (0.1 to 0.7) |  |
| First infant per woman, valproic acid vs. lamotrigine | 660 / 1,576 | 0.1 (-0.1 to 0.4) | -0.3 (-0.8 to 0.1) | 0.0 (-0.3 to 0.3) | 0.5 (0.1 to 1.0) |  |
| Female infants, valproic acid vs. lamotrigine | 472 / 1,091 | 0.0 (-0.3 to 0.4) | -0.3 (-0.9 to 0.4) | 0.0 (-0.2 to 0.2) | 0.1 (-0.3 to 0.5) |  |
| Male infants, valproic acid vs. lamotrigine | 494 / 992 | 0.2 (-0.2 to 0.5) | -0.2 (-0.9 to 0.5) | 0.5 (0.2 to 0.9) | 0.8 (0.2 to 1.3) |  |
| High vs. low dose of valproic acid | 167 / 167 | -0.0 (-0.8 to 0.8) | -0.4 (-2.5 to 1.7) | 0.0 (-0.7 to 0.7) | 1.2 (0.2 to 2.1) |  |
| **Birth head circumference (cm)** | | | | | | |
| Use any time in pregnancy, valproic acid vs. lamotrigine | 931 / 2,059 | -0.2 (-0.3 to -0.0) | -0.0 (-0.3 to 0.3) | -0.0 (-0.0 to 0.0) | -0.0 (-0.1 to 0.1) |  |
| Use in first trimester, valproic acid vs. lamotrigine | 802 / 1,877 | -0.2 (-0.4 to -0.1) | -0.0 (-0.3 to 0.3) | -0.0 (-0.0 to 0.0) | -0.0 (-0.1 to 0.1) |  |
| Continuers, valproic acid vs. lamotrigine | 252 / 983 | -0.4 (-0.7 to -0.2) | -0.9 (-1.4 to -0.4) | -0.0 (-0.3 to 0.2) | 0.0 (-0.4 to 0.4) |  |
| Mother with epilepsy, valproic acid vs. lamotrigine | 759 / 1,395 | -0.2 (-0.4 to -0.1) | -0.4 (-0.8 to -0.1) | -0.0 (-0.0 to 0.0) | -0.0 (-0.2 to 0.2) |  |
| Mother with chronic pain, valproic acid vs. lamotrigine | 140 / 537 | 0.0 (-0.3 to 0.4) | -0.5 (-1.4 to 0.4) | 0.0 (-0.4 to 0.4) | 0.0 (-0.6 to 0.6) |  |
| Monotherapy, valproic acid vs. lamotrigine | 824 / 1,767 | -0.1 (-0.3 to 0.1) | -0.2 (-0.5 to 0.1) | -0.0 (-0.0 to 0.0) | -0.0 (-0.1 to 0.1) |  |
| Polytherapy, valproic acid vs. lamotrigine | 107 / 292 | -0.8 (-1.2 to -0.4) | -1.0 (-2.1 to 0.0) | -0.4 (-0.8 to 0.0) | -0.5 (-1.1 to 0.1) |  |
| Definite exposure, valproic acid vs. lamotrigine | 266 / 1,040 | -0.4 (-0.7 to -0.1) | -0.8 (-1.4 to -0.2) | -0.0 (-0.1 to 0.1) | -0.1 (-0.5 to 0.2) |  |
| Complete-case analysis, valproic acid vs. lamotrigine | 821 / 1,853 | -0.2 (-0.4 to -0.1) | -0.3 (-0.6 to -0.0) | -0.0 (-0.0 to 0.0) | -0.0 (-0.1 to 0.1) |  |
| Singletons with no MCMs, valproic acid vs. lamotrigine | 770 / 1,848 | -0.1 (-0.3 to 0.0) | -0.2 (-0.4 to 0.0) | -0.0 (-0.0 to 0.0) | -0.0 (-0.2 to 0.2) |  |
| First infant per woman, valproic acid vs. lamotrigine | 634 / 1,559 | -0.2 (-0.4 to -0.0) | -0.3 (-0.7 to 0.1) | -0.0 (-0.1 to 0.0) | -0.0 (-0.3 to 0.2) |  |
| Female infants, valproic acid vs. lamotrigine | 458 / 1,078 | -0.2 (-0.4 to 0.0) | -0.5 (-0.9 to -0.1) | -0.2 (-0.4 to 0.1) | -0.1 (-0.3 to 0.1) |  |
| Male infants, valproic acid vs. lamotrigine | 473 / 981 | -0.2 (-0.4 to -0.0) | -0.3 (-0.7 to 0.1) | -0.0 (-0.2 to 0.2) | -0.1 (-0.4 to 0.2) |  |
| High vs. low dose of valproic acid | 166 / 162 | -0.6 (-1.1 to -0.1) | -1.1 (-2.3 to 0.0) | -0.0 (-0.4 to 0.4) | 0.2 (-0.7 to 1.0) |  |

AED = antiepileptic drug; CI = confidence interval; MCM = major congenital malformation; SGA = small for gestational age.

AED use was ascertained at any time in pregnancy, except where noted (indented rows). Analyses on continuers used data from deliveries in 2006-2013. In analyses of carbamazepine vs. lamotrigine, the reference was lamotrigine in the same exposure window. In dose-response analyses, the reference was the bottom tertile of mean daily dose of carbamazepine (2006-2013). To facilitate convergence of quantile regression models for birth length and head circumference in cm, we implemented 1% jittering of birth length and head circumference by introducing random noise with uniform distribution and width 0.01 around the observation’s value. All results were adjusted for birth year, maternal age at delivery, education, country of origin, marital status, body mass index, smoking in current pregnancy, alcohol dependence, diabetes, hypertension, epilepsy, depression, bipolar disorder, migraine, chronic pain, and other psychiatric disorders. When the smallest cell count was < 5, we did not produce adjusted results ("not applicable"). Models restricted to polytherapy compared infants exposed to carbamazepine and another AED (except lamotrigine) with those exposed to lamotrigine and another AED (except carbamazepine).

1. Effect-measure modification analysis for the association between in-utero valproic acid exposure and the endpoints duration of pregnancy and size at birth

|  | | | **Adjusted difference (95% CI)** | | |  |
| --- | --- | --- | --- | --- | --- | --- |
| **Potential EMM** | **Endpoint** | **Exposed to valproic acid / lamotrigine** | **Valproic acid** | **Potential EMM** | **Interaction term** | **P-value for interaction term** |
| Smoking in pregnancy | Pregnancy duration (days) | 985 / 2,086 | 0.5 (-0.8 to 1.9) | -1.2 (-3.0 to 0.6) | -3.1 (-6.1 to -0.2) | 0.036 |
|  | Birth weight z-score | 992 / 2,110 | -0.1 (-0.2 to 0.0) | -0.4 (-0.6 to -0.3) | 0.0 (-0.2 to 0.3) | 0.670 |
|  | Birth length z-score | 966 / 2,083 | 0.1 (-0.0 to 0.2) | -0.4 (-0.5 to -0.3) | 0.1 (-0.1 to 0.3) | 0.426 |
|  | Head circumference z-score | 931 / 2,059 | -0.2 (-0.3 to -0.1) | -0.3 (-0.5 to -0.2) | 0.1 (-0.1 to 0.3) | 0.312 |
|  | Birth weight (grams) | 992 / 2,110 | -15 (-71 to 40) | -177 (-253 to -101) | -70 (-193 to 53) | 0.265 |
|  | Birth length (cm) | 966 / 2,083 | 0.1 (-0.1 to 0.4) | -0.8 (-1.1 to -0.5) | -0.1 (-0.7 to 0.5) | 0.707 |
|  | Head circumference (cm) | 931 / 2,059 | -0.2 (-0.4 to -0.0) | -0.4 (-0.6 to -0.2) | 0.0 (-0.4 to 0.4) | 0.992 |
| Use of SSRIs in pregnancy | Pregnancy duration (days) | 985 / 2,086 | 0.4 (-0.9 to 1.7) | -1.8 (-3.8 to 0.2) | -3.9 (-7.7 to -0.1) | 0.043 |
|  | Birth weight z-score | 992 / 2,110 | -0.0 (-0.1 to 0.1) | -0.1 (-0.2 to 0.1) | -0.0 (-0.3 to 0.3) | 0.826 |
|  | Birth length z-score | 966 / 2,083 | 0.1 (0.0 to 0.2) | -0.2 (-0.4 to -0.1) | -0.1 (-0.3 to 0.2) | 0.701 |
|  | Head circumference z-score | 931 / 2,059 | -0.2 (-0.2 to -0.1) | -0.1 (-0.2 to 0.1) | 0.0 (-0.3 to 0.3) | 0.920 |
|  | Birth weight (grams) | 992 / 2,110 | -15 (-68 to 38) | -48 (-131 to 34) | -123 (-283 to 36) | 0.130 |
|  | Birth length (cm) | 966 / 2,083 | 0.2 (-0.1 to 0.4) | -0.5 (-0.9 to -0.1) | -0.4 (-1.2 to 0.3) | 0.234 |
|  | Head circumference (cm) | 931 / 2,059 | -0.2 (-0.3 to -0.0) | -0.1 (-0.3 to 0.1) | -0.3 (-0.8 to 0.2) | 0.232 |

AED = antiepileptic drug; CI = confidence interval; EMM = effect-measure modifier; SSRI = selective serotonin reuptake inhibitor.

AED use was ascertained at any time in pregnancy. The reference was lamotrigine in the same exposure window. Results were obtained with linear regression analysis models and were adjusted for birth year, maternal age at delivery, education, country of origin, marital status, body mass index, smoking in current pregnancy, alcohol dependence, diabetes, hypertension, epilepsy, depression, bipolar disorder, migraine, chronic pain, and other psychiatric disorders.

1. Association between in-utero lamotrigine exposure and the endpoints duration of pregnancy and size at birth

|  | | **Difference (95% CI)** | | | | **Odds ratio** |
| --- | --- | --- | --- | --- | --- | --- |
|  | | | **At percentile** | | | **(95% CI)** |
|  | **Exposed to lamotrigine/ reference** | **Mean** | **10^th^** | **50^th^** | **90^th^** |  |
| **Pregnancy duration (days)                                     Preterm birth** | | | | | | |
| High vs. low dose of lamotrigine | 551 / 547 | -1.8 (-3.8 to 0.2) | -0.9 (-5.1 to 3.3) | -0.6 (-2.6 to 1.3) | -1.1 (-3.2 to 1.0) | 1.3 (0.7 to 2.2) |
| **Birth weight z-score                                                       SGA** | | | | | | |
| High vs. low dose of lamotrigine | 557 / 557 | 0.1 (-0.1 to 0.2) | -0.0 (-0.3 to 0.2) | 0.1 (-0.0 to 0.3) | 0.1 (-0.1 to 0.3) | 0.9 (0.3 to 2.1) |
| **Birth length z-score** | | | | | | |
| High vs. low dose of lamotrigine | 548 / 551 | 0.1 (-0.1 to 0.2) | -0.1 (-0.3 to 0.2) | -0.0 (-0.2 to 0.1) | 0.3 (0.1 to 0.5) |  |
| **Birth head circumference z-score                                    Microcephaly** | | | | | | |
| High vs. low dose of lamotrigine | 543 / 550 | 0.0 (-0.1 to 0.2) | -0.1 (-0.4 to 0.1) | -0.0 (-0.2 to 0.2) | 0.0 (-0.2 to 0.3) | 0.5 (0.2 to 1.6) |
| **Birth weight (grams)** | | | | | | |
| High vs. low dose of lamotrigine | 557 / 557 | -11 (-95 to 73) | -100 (-245 to 46) | 33 (-52 to 118) | 10 (-120 to 140) |  |
| **Birth length (cm)** | | | | | | |
| High vs. low dose of lamotrigine | 548 / 551 | 0.1 (-0.3 to 0.4) | 0.0 (-0.7 to 0.7) | -0.0 (-0.4 to 0.4) | 0.4 (-0.1 to 1.0) |  |
| **Birth head circumference (cm)** | | | | | | |
| High vs. low dose of lamotrigine | 543 / 550 | -0.0 (-0.3 to 0.3) | 0.0 (-0.4 to 0.4) | 0.0 (-0.0 to 0.0) | 0.1 (-0.3 to 0.4) |  |

AED = antiepileptic drug; CI = confidence interval; MCM = major congenital malformation; SGA = small for gestational age.

AED use was ascertained at any time in pregnancy. The reference was the bottom tertile of mean daily dose of lamotrigine (2006-2013). To facilitate convergence of quantile regression models for birth length and head circumference in cm, we implemented 1% jittering of birth length and head circumference by introducing random noise with uniform distribution and width 0.01 around the observation’s value. Results were adjusted for birth year, maternal age at delivery, education, country of origin, marital status, body mass index, smoking in current pregnancy, alcohol dependence, diabetes, hypertension, epilepsy, depression, bipolar disorder, migraine, chronic pain, and other psychiatric disorders.

1. Estimated effects from linear regression models for key variables

|  | **Estimated effect (95% confidence interval)** | | | |
| --- | --- | --- | --- | --- |
| **Endpoint** | **Antiepileptic drug** | **Smoking** | **Epilepsy** | **Diabetes** |
| **Carbamazepine** | | | | |
| Pregnancy duration (days) | -1.3 (-2.3 to -0.3) | -1.7 (-3.1 to -0.3) | -0.7 (-2.1 to 0.6) | -7.8 (-10.4 to -5.2) |
| Birth weight z-score | -0.1 (-0.2 to -0.0) | -0.5 (-0.6 to -0.4) | -0.0 (-0.1 to 0.1) | 0.8 (0.6 to 0.9) |
| Birth length z-score | -0.1 (-0.2 to -0.0) | -0.4 (-0.5 to -0.3) | -0.0 (-0.1 to 0.1) | 0.4 (0.2 to 0.6) |
| Head circumference z-score | -0.2 (-0.3 to -0.1) | -0.3 (-0.4 to -0.2) | -0.0 (-0.1 to 0.1) | 0.3 (0.1 to 0.5) |
| **Levetiracetam** | | | | |
| Pregnancy duration (days) | -0.5 (-2.6 to 1.6) | -1.2 (-2.9 to 0.5) | 0.5 (-1.5 to 2.4) | -8.7 (-11.9 to -5.4) |
| Birth weight z-score | -0.1 (-0.3 to 0.0) | -0.5 (-0.6 to -0.3) | 0.0 (-0.1 to 0.2) | 0.7 (0.5 to 1.0) |
| Birth length z-score | -0.0 (-0.1 to 0.1) | -0.5 (-0.6 to -0.3) | 0.0 (-0.1 to 0.2) | 0.3 (0.1 to 0.5) |
| Head circumference z-score | -0.1 (-0.3 to 0.1) | -0.4 (-0.5 to -0.2) | 0.0 (-0.1 to 0.1) | 0.3 (0.0 to 0.5) |
| **Pregabalin** | | | | |
| Pregnancy duration (days) | -1.1 (-3.0 to 0.8) | -1.2 (-2.7 to 0.3) | -0.5 (-2.3 to 1.2) | -8.9 (-11.9 to -5.9) |
| Birth weight z-score | -0.1 (-0.3 to 0.0) | -0.5 (-0.6 to -0.4) | 0.0 (-0.1 to 0.1) | 0.9 (0.7 to 1.1) |
| Birth length z-score | -0.1 (-0.2 to 0.0) | -0.5 (-0.6 to -0.4) | -0.0 (-0.1 to 0.1) | 0.5 (0.3 to 0.7) |
| Head circumference z-score | -0.0 (-0.1 to 0.1) | -0.4 (-0.5 to -0.3) | -0.0 (-0.2 to 0.1) | 0.4 (0.2 to 0.6) |
| **Valproic acid** | | | | |
| Pregnancy duration (days) | -0.0 (-1.2 to 1.2) | -2.3 (-3.8 to -0.8) | -1.6 (-3.3 to 0.1) | -8.2 (-11.1 to -5.3) |
| Birth weight z-score | -0.0 (-0.1 to 0.0) | -0.4 (-0.5 to -0.3) | 0.0 (-0.1 to 0.1) | 0.8 (0.6 to 1.0) |
| Birth length z-score | 0.1 (0.0 to 0.2) | -0.4 (-0.5 to -0.3) | 0.0 (-0.1 to 0.1) | 0.4 (0.2 to 0.6) |
| Head circumference z-score | -0.2 (-0.2 to -0.1) | -0.3 (-0.4 to -0.2) | -0.0 (-0.1 to 0.1) | 0.4 (0.2 to 0.6) |

These are estimates from linear regression models for antiepileptic drug exposure ascertained at any time in pregnancy. The reference is lamotrigine in the same time window. All results are adjusted for birth year, maternal age at delivery, education, country of origin, marital status, body mass index, smoking in current pregnancy, alcohol dependence, diabetes, hypertension, epilepsy, depression, bipolar disorder, migraine, chronic pain, and other psychiatric disorders.
